# Novel Curcumin-Diethyl Fumarate Hybrid as a Dualistic GSK-3β Inhibitor/Nrf2 Inducer for the Treatment of Parkinson’s Disease

**DOI:** 10.1021/acschemneuro.0c00363

**Published:** 2020-07-14

**Authors:** Rita Maria Concetta Di Martino, Letizia Pruccoli, Alessandra Bisi, Silvia Gobbi, Angela Rampa, Ana Martinez, Concepción Pérez, Loreto Martinez-Gonzalez, Maria Paglione, Elia Di Schiavi, Francesca Seghetti, Andrea Tarozzi, Federica Belluti

**Affiliations:** † Department of Pharmacy and Biotechnology, Alma Mater Studiorum - University of Bologna, Via Belmeloro 6, 40126 Bologna, Italy; ‡ Department for Life Quality Studies, Alma Mater Studiorum - University of Bologna, Corso d’Augusto 237, 47921 Rimini, Italy; ∥ Centro de Investigaciones Biologica, CSIC, Ramiro de Maeztu 9, 28040 Madrid, Spain; # Department of Biology, Agriculture and Food Science, National Research Council (CNR), Institute of Biosciences and BioResources (IBBR), Via Pietro Castellino 111, 80131 Naples, Italy

**Keywords:** Curcumin analogues, Diethyl fumarate, Neurodegeneration, Nuclear factor-erythroid related factor 2, Glycogen synthase kinase-3β, Oxidative stress, Parkinson’s disease

## Abstract

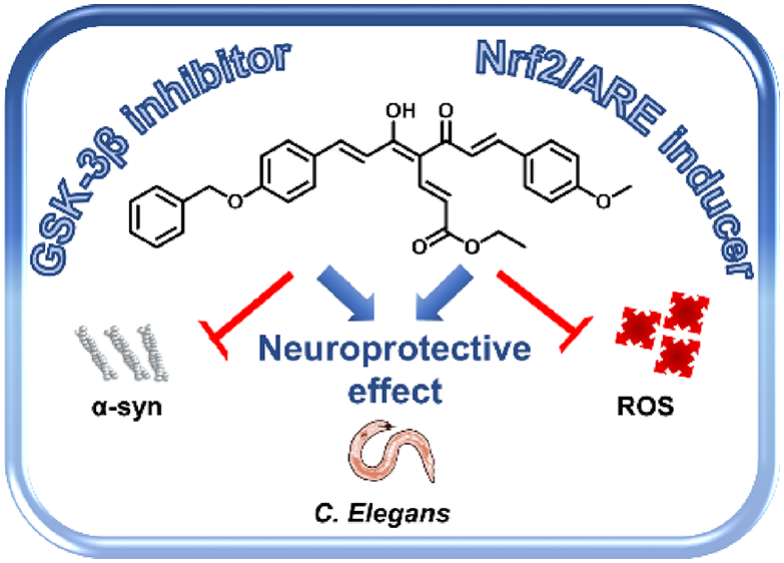

Common copathogenic factors, including oxidative stress and neuroinflammation, are found to play a vital role in the development of neurodegenerative disorders, including Alzheimer’s disease (AD) and Parkinson’s disease (PD). Nowadays, owing to the multifactorial character of the diseases, no effective therapies are available, thus underlying the need for new strategies. Overexpression of the enzyme GSK-3β and downregulation of the Nrf2/ARE pathway are responsible for a decrease in antioxidant defense effects. These pieces of evidence underline the usefulness of dual GSK-3β inhibitors/Nrf2 inducers. In this regard, to design a dual modulator, the structures of a curcumin-based analogue, as GSK-3β inhibitor, and a diethyl fumarate fragment, as Nrf2 inducer, were combined. Among the hybrids, **5** and **6** proved to effectively inhibit GSK-3β, while **4** and **5** showed a marked ability to activate Nrf2 together to increase the neuronal resistance to oxidative stress. These last pieces of evidence translated into specific neuroprotective effects of **4** and **5** against PD pathological events including neurotoxicity elicited by α-synuclein aggregates and 6-hydroxydopamine. Hybrid **5** also showed neuroprotective effects in a *C. elegans* model of PD where the activation of GSK-3β is intimately involved in Nrf2 regulation. In summary, **5** emerged as an interesting multitarget derivative, valuable to be exploited in a multitarget PD perspective.

## Introduction

Neurodegenerative diseases (NDDs) such as Alzheimer’s disease (AD), Huntington’s disease, Parkinson’s disease (PD), and amyotrophic lateral sclerosis are increasingly recognized as major causes of death and disability worldwide.^1^ They are characterized by a progressive impairment of cognitive and/or motor functions that reflect the loss of specific neurons in distinct central nervous system (CNS) regions.^2^

Although AD and PD, the two most common NDDs, show different clinical profiles, common molecular pathogenic mechanisms including oxidative stress, proteostasis, mitochondrial deficit, glutamate excitotoxicity, and neuroinflammation are observed in both diseases, suggesting converging pathways of neurodegeneration.^3^

In particular, oxidative stress is implicated in the proteostasis phenomena and leads to misfolding of neurotoxic proteins such as α-synuclein (α-syn) in Lewy bodies (LBs) of PD and phospho-tau (p-tau) and amyloid-β (Aβ) in neurofibrillary tangles and senile plaques, respectively, of AD.^4^

In this regard, there are several strategies for reducing the toxic effects of α-syn and p-tau targeting the different proteostasis pathways.^5^ Among the shared pathogenetic pathways, the dysregulation of the glycogen synthase kinase-3β (GSK-3β)/nuclear factor-erythroid related factor 2 (Nrf2) signaling pathway is implicated in the oxidative stress defenses in both AD and PD.^6^ Nrf2 plays an important role in antioxidant mechanisms in response to oxidative stress: in basal conditions, it is bound to its endogenous inhibitor Kelch-like ECH associated protein1 (Keap1), a cysteine-rich zinc–metalloprotein, involved in promoting Nrf2 degradation. In response to stress insults, such as reactive oxygen species (ROS), this factor is released from Keap1, and upon translocation to the nucleus, it binds to the antioxidant response element (ARE). The Keap1/Nrf2/ARE complex directly regulates the expression of some phase II detoxifying enzymes and antioxidant stress genes, namely NAD(P)H: quinone oxidoreductase 1 (NQO1), heme oxygenase-1, glutathione S-transferase, and aldo-keto reductase.^7^ Further, Nrf2 ameliorates the inflammation response by inhibiting the translocation of nuclear factor-kB (NF-kB) and activating anti-inflammatory genes.^8,9^ An altered expression of Nrf2 in both neurons and astrocytes of PD and AD patients has been observed. More recent studies suggest that Nrf2 activation reduces α-syn and p-tau levels, facilitating the degradation of these toxic proteins through autophagy.^10−12^ Thus, the Nrf2 signaling cascade, as a valuable defense against oxidative stress insults and loss of proteostasis, has been recognized as a validated target for the development of therapeutics for PD and AD treatment.

The enzyme GSK-3, a multifunctional serine/threonine kinase, exists in two isoforms, i.e., GSK-3α and GSK-3β, and participates in a wide range of cellular processes and signaling pathways; its increased expression and activity have been observed in several NDDs including AD and PD. In particular, GSK-3β, represents a determining factor for abnormal tau protein phosphorylation and aggregation into neurofibrillary tangles. In PD, elevated levels of hyperphosphorylated tau were also associated with high levels of insoluble α-syn, underlying the pivotal role of the α-syn/p-tau/GSK-3β pathway. These events culminated in extensive oxidative stress and neuronal cell death.^13^ Regarding the functional aspects, this kinase is regulated by post-translational phosphorylations at Ser9 and Tyr216, associated with enzyme inhibition and activation, respectively. Moreover, activated GSK-3β plays a pivotal role in the downregulation of Nrf2 through direct phosphorylation of Nrf2 that, in turn, promotes proteasomal degradation of Nrf2 in a Keap1-independent manner.^14^ Definitively, the inverse correlation between the aberrant activation of GSK-3β and the downregulation of Nrf2, with a corresponding decrease of antioxidant gene expression and cell defense effects, reinforces the usefulness of GSK-3β inhibition in achieving therapeutic benefit.^4^

The complex pathological mechanisms characterizing NNDs prompted consideration of polypharmacology as the most appropriate therapeutic approach by which to accomplish an effective and disease-modifying outcome. In particular, by modulating several targets involved in pathology, multitarget drugs could offer the possibility of attaining improved effectiveness compared to a single-target drug.^15,16^ In this complex framework, the negative correlation between GSK-3β and Nrf2 suggests that small molecules joining GSK-3β inhibition to Nrf2 induction could be valuable drug candidates for NDDs.^17^ The modulation of these targets, involved in the same signaling pathways in a feed-forward manner, could likely make it possible to obtain superior beneficial effect by reducing possible compensatory mechanisms and eliciting synergistic outcomes. Identifying chemical tools able to treat oxidative stress and inflammation, through the concomitant modulation of Nrf2 and GSk-3β, could allow shedding light into this crucial cross talk.

**Figure 1:**
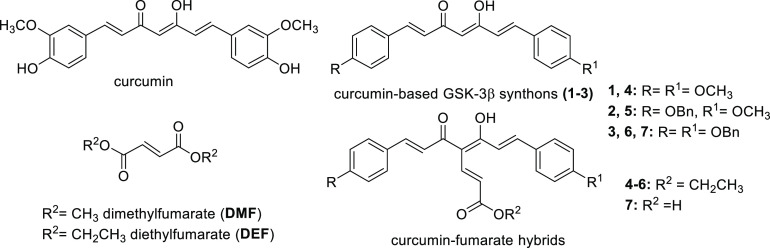
Chemical structures of curcumin and general structures of curcumin-based starting synthons (**1**–**3**), fumaric acid esters (DMF and DEF), and curcumin-fumarate hybrids (**4**–**7**).

Curcumin (Figure 1), the most abundant component of *Curcuma longa* rhizome, has gained considerable interest due to its pleiotropic therapeutic profile.^18^ At the molecular level, naturally occurring curcuminoids proved to modulate a large variety of interconnected pathways implicated in the pathogenesis of several multifactorial diseases such as cancer and NDDs.^19^ The capability of regulating the mediators and biological targets of the inflammation cascade contributes to this therapeutic effect.^20^ In this respect, this natural compound, regarded as privileged structure, furnished the scaffold for the design of a large number of functionalized curcumin-based analogues endowed with significant antineurodegenerative activities, thus corroborating the enormous potential of this template in drug discovery.^21^

Several drawbacks have been reported for curcumin including poor solubility and chemical and metabolic instability that, in turn, negatively affect pharmacokinetic/pharmacodynamic properties, mainly ascribed to the 3-methoxy-4-hydroxy substitution pattern of the side aryl moieties. Moreover, curcumin and a variety of biologically active natural compounds, among them epigallocatechin gallate, mitoxantrone, resveratrol, and rifampicin, have been classified as pan-assay interfering compounds (PAINS) as they can generate false assay signals.^22,18^ Nevertheless, it is a widely shared opinion that compound reactivity might be dependent on the structural context; thus, properly designed molecules containing a PAINS-based structural motif can behave as selective modulators of some well-defined targets rather than reactive chemicals.^23,24^ This statement is strongly sustained by a large number of publications reporting bioactive curcumin-related molecules obtained upon adequate modification of the main framework, as documented by review articles.^21c^

## Results and Discussion

### Design Strategy of Curcumin-DEF Hybrids as Dual GSK-3β/Nrf2 Modulators

GSK-3β and Nrf2 could be regarded as valuable targets for NDDs treatment; their concurrent modulation through multitarget agents could offer promises for obtaining an improved therapeutic outcome associated with fewer undesirable side effects. Thus, the design of chemical entities endowed with the capability to concurrently interact with Nrf2 and GSK-3β is a challenging task.

Several natural compounds endowed with an electrophilic motif, a well-established Michael acceptor system, proved to activate the Nrf2 pathway;^25^ in particular, curcumin and fumaric acid esters (FAEs), i.e., dimethyl fumarate (DMF), were reported to covalently bind crucial cysteine residues of Keap1.^26^ In a previous study, aimed at identifying new disease-modifying AD drug candidates, a small library of curcumin-based analogues was developed, leading to the identification of some active and balanced dualistic inhibitors of the enzymes β-secretase (BACE-1) and GSK-3β also able to induce the antioxidant and anti-inflammatory enzyme NQO1. In line with this, aimed at designing new chemical entities as dual Nrf2 and GSK-3β modulators, the previously identified GSK-3β inhibitors **1**–**3** (Figure 1, IC_50_ = 0.53, 2.78, and 2.49 μM, respectively), chemically characterized by combinations of benzyloxy and methoxy substituents on the side aryl functions of the curcumin scaffold,^21b^ were selected as the starting platform for a hybridization strategy consisting of the introduction of a diethyl fumarate (DEF) fragment at the 4-position of the heptadienone framework.^27^

### Chemistry

**Scheme 1:**
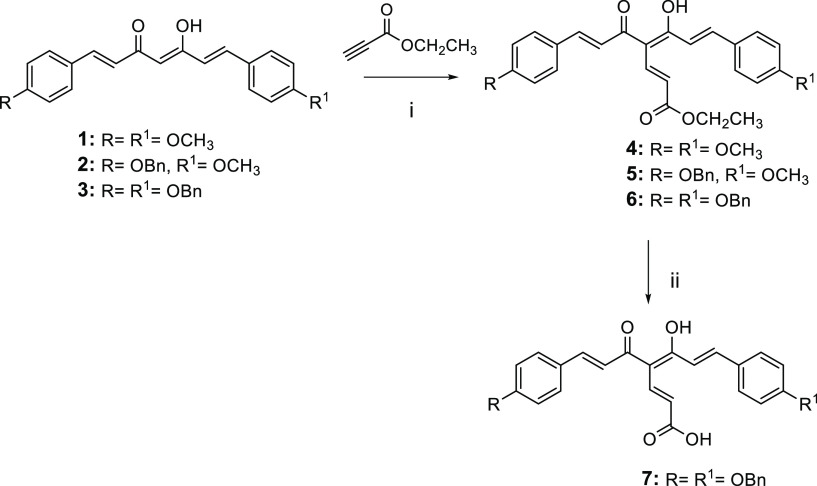
Reagents and conditions: (i) NaH, dry THF, 0 °C to rt, N_2_, overnight, 41–84% yield; (ii) KOH (2 N CH_3_OH solution), CH_3_OH, 60 °C, 12 h, 75% yield.

The synthetic strategy for obtaining the curcumin-fumarate hybrids (**4**–**7**) is outlined in Scheme 1. The reaction of the starting synthons **1**–**3** with ethylpropiolate by using NaH as a base gave the corresponding analogues **4**–**6**. The ethyl ester function of **6** was converted into an acidic function by treatment with KOH, to obtain **7**.

### Biological Evaluations

The newly synthesized compounds (**4**–**7**) were studied for their anti-NDD potential, namely the capability to affect Nrf2 and GSK-3β activities, along with the protective effect against a neurotoxic insult. In this context, a screening pipeline was followed. First, their inhibition of the GSK-3β enzyme was evaluated by using a Luminescent Kinase Assay based on the quantification of the amount of the ATP present after the kinase reaction. In parallel, the neurotoxic effects of compounds **4**–**7** were evaluated in human neuronal cells (SH-SY5Y) in order to define the range of concentrations to be employed in the experimental setting. The most promising compounds were studied for their indirect antioxidant effect (ability to modulate the Keap1/Nrf2/ARE pathway) upon assessment of their capability to induce glutathione (GSH) release and to decrease intracellular ROS formation under oxidative stress. In this regard, we used DMF as a positive control due to its known ability to mediate the antioxidant cellular defense by Nrf2 activation.^26^ Afterward, analogues endowed with this property were selected to be evaluated for Nrf2 activation in terms of translocation of Nrf2 from the cytosol to the nucleus, Nrf2/ARE binding, and transcriptional activity. To achieve critical evidence regarding the therapeutic context of the tested compounds, namely AD or PD, studies on the protective effects against specific toxic insults by Aβ_1–42_ oligomers and 6-hydroxydopamine (6-OHDA) were performed. The effect of **4** and **5**, characterized, among the series, by the more potent counteracting effects against 6-OHDA stimulus, was also investigated for their effect on α-syn aggregates, to confirm their anti-PD potential. Moreover, the neuroprotective activity of these compounds was also verified in a *C. elegans* assay, a well-validated PD animal model, by observing the neurodegeneration of the cephalic (CEP) neurons. Finally, the ability to cross the blood-brain barrier (BBB), an essential feature for achieving a CNS effect, was preliminarily investigated for all the hybrids by means of an *in vitro* parallel artificial membrane permeability (PAMPA)-BBB assay.

Moreover, to gain insight on one of the possible underlying mechanisms of action, the GSK-3α/β (Ser21/9) kinase activity was assessed for the most potent and interesting derivative **5** (see Figure S1, Supporting Information).

### GSK-3β Inhibition

**Figure 2:**
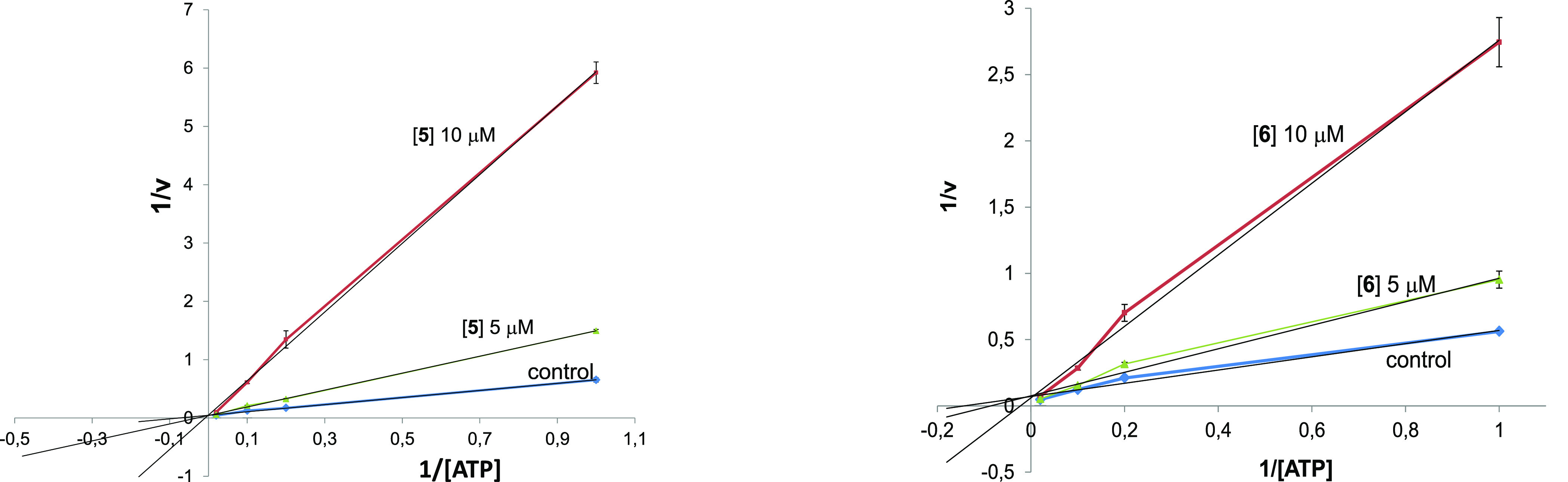
Kinetic data for the curcumin-DEF hybrids **5** and **6**. In the reaction mixture, the concentration of compounds employed is reported in the plot, the concentration of the substrate is constant (25 μM), and the concentration of ATP changes from 1 to 50 μM. Each point is the mean of two different experiments analyzed in triplicate.

A luminescence method was employed to assess the GSK-3β inhibitory effect of the synthesized compounds by using a human recombinant enzyme.^28^ The compounds **4**–**7** were first tested at the highest 10 μM concentration, then, for derivatives showing an inhibition percentage over 50%, the IC_50_ value was determined by performing a linear regression analysis and using 4-benzyl-2-methyl-1,2,4-thiadiazolidine-3,5-dione as the reference compound.^29^ While **4** and **7** when tested at 10 μM concentration showed a low GSK-3β inhibitory effect (40% and 46%, respectively), for **5** and **6** low micromolar IC_50_ values of 8.4 and 6.1 μM, respectively, were recorded (Table 1). This result could be exciting, since a smooth therapeutic inhibition of GSK-3β, allowing for CNS upregulated enzyme levels to return to physiological levels while not affecting normal peripheral enzyme levels, has been indicated as a safe approach.^30^ Thus, the mechanism of inhibition as regards competition with ATP was investigated for **5** and **6**. A kinetic study was performed in which the concentrations of both ATP and tested compounds were varied, while the concentration of the substrate was kept constant. By observing the graphs (Figure 2) reporting the double reciprocal plot of the data, we can speculate that **5** and **6** act as noncompetitive inhibitors of ATP binding, as by increasing concentration of ATP from 1 to 50 μM, enzymatic inhibition is not affected.

**Table 1:**
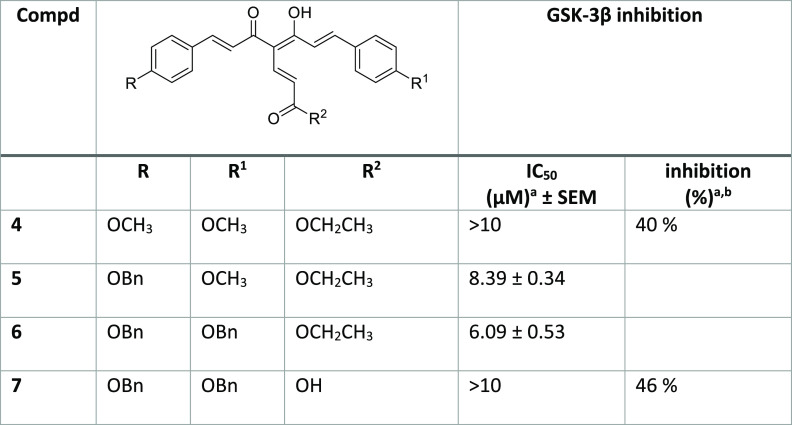
Inhibition of GSK-3β Enzymatic Activity by the Curcumin-Fumarate Hybrids (**4**–**7**)

a Values are mean ± SD of two independent measurements, each performed in triplicate. SEM = standard error of the mean.

b Inhibition % of GSK-3β activity at the concentration of 10 μM of the tested compounds.

### Cytotoxicity

The 3-(4,5-dimethylthiazol-2-yl)-2,5-diphenyltetrazolium bromide (MTT) assay was used to evaluate the cytotoxic effects of the compounds. The SH-SY5Y cells were exposed to compound concentrations ranging from 1.25 to 40 μM for 24 h, and cell viability was evaluated by MTT. The treatment of SH-SY5Y cells with all the tested compounds at the concentrations lower than 10 μM did not affect cell viability (see Figure S9, Supporting Information). Thereby, the concentration of 5 μM was selected to perform all the following assays in SH-SY5Y.

### Antioxidant Activity

**Figure 3:**
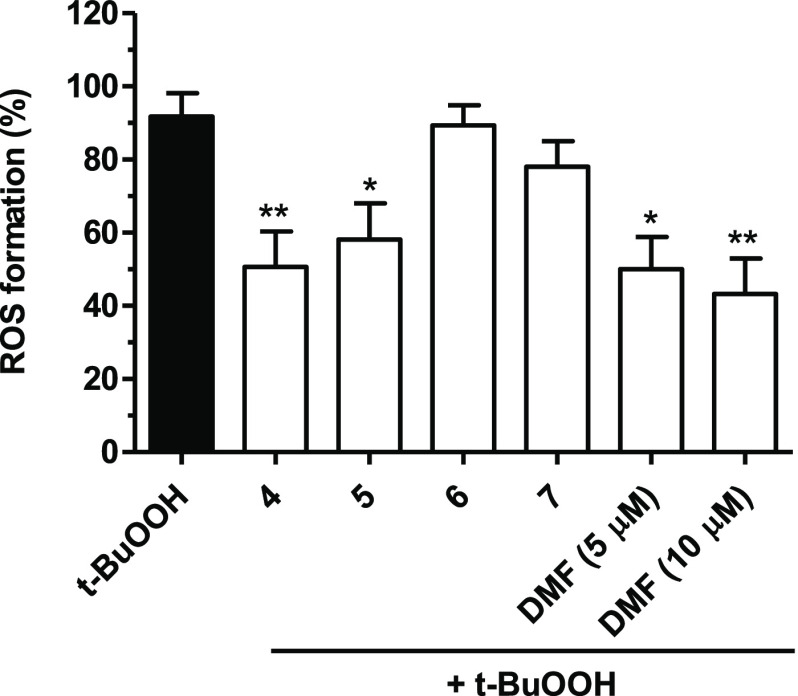
Antioxidant activity of compounds **4**–**7** and DMF against *t*-BuOOH-induced ROS formation in SH-SY5Y cells. Cells were treated with compounds **4**–**7** (5 μM) and DMF (5 and 10 μM) for 24 h and then with *t-*BuOOH (100 μM) for 30 min. At the end of treatment, the ROS formation was evaluated by probe H_2_DCF-DA. Results are expressed as mean ± SEM of at least three independent experiments (* *p* < 0.05 and ** *p* < 0.01 vs cells treated with *t*-BuOOH at one-way ANOVA with the Dunnett post hoc test).

Oxidative stress represents a remarkable pathological feature in NDDs; it is the consequence of an imbalance between the formation of reactive radical species, among others ROS, and the function of many defense antioxidant enzymes. Initially, the potential of compounds **4**–**7** as indirect antioxidants was investigated^31^ by assessing their capability to protect from oxidative stress through prevention of ROS formation upon exposure to *tert*-butyl hydroperoxide (*t*-BuOOH). In detail, SH-SY5Y cells were first chronically treated for 24 h (the time necessary to activate the endogenous antioxidant system) with **4**–**7** (5 μM) and then exposed to 100 μM of *t*-BuOOH for 30 min; DMF (5 and 10 μM) was employed as the reference compound. Then, the intracellular ROS formation was detected by fluorescent probe 2′-7′-dichlorodihydrofluorescein diacetate (H_2_DCF-DA). The results, depicted in Figure 3, show a significant reduction in *t*-BuOOH-induced intracellular ROS formation after the treatment with compounds **4** and **5** and DMF but not with **6** and **7**.

**Figure 4:**
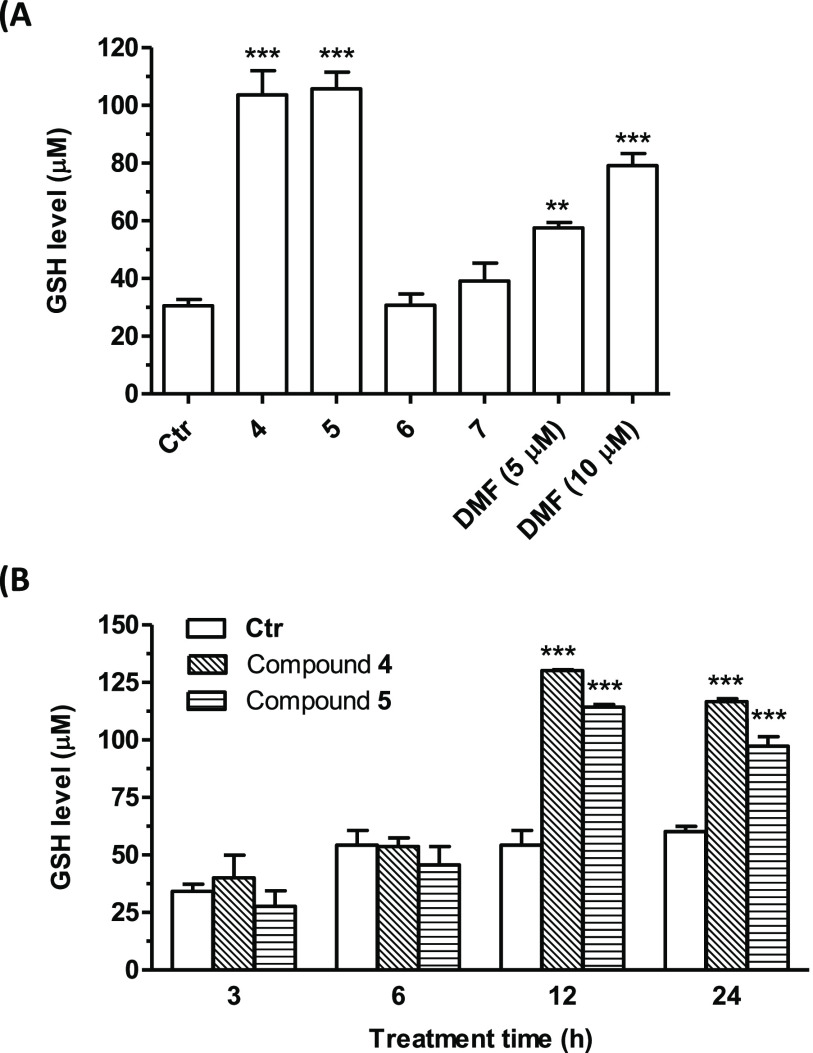
Effects of compounds **4**–**7** and DMF on GSH levels in SH-SY5Y cells. Cells were treated with (A) compounds **4**–**7** (5 μM) and DMF (5 and 10 μM) for 24 h. (B) Cells were treated with compounds **4** and **5** (5 μM) for different times (3, 6, 12, and 24 h). At the end of the treatment, the GSH levels were evaluated by probe MCB. Results are expressed as mean ± SEM of at least three independent experiments (** *p* < 0.01 and *** *p* < 0.001 vs untreated cells at one-way ANOVA with the Dunnett post hoc test).

By observing the reduction of ROS levels following a long treatment of the curcumin-DEF hybrids before the treatment with *t*-BuOOH, we hypothesized that the antioxidant effect might likely result from an increase in levels of GSH. This endogenous antioxidant plays critical roles in protecting cells against oxidative stress damage.^32^ Thus, the intracellular GSH levels were analyzed by employing the same experimental conditions used to evaluate the indirect antioxidant effect by fluorescent probe monochlorobimane (MCB). Treatment of SH-SY5Y cells with 5 μM of compounds **4**–**7** determined an increase in intracellular GSH levels (Figure 4A). The most active compounds were **4** and **5**, with a 4-fold increase in GSH levels as compared to control. These effects were significantly higher than those elicited by DMF, a well-known inducer of GSH biosynthesis for which 2–3-fold increases were observed when tested at concentrations of 5 and 10 μM (Figure 4A). For the most promising compounds **4** and **5**, the effect on GSH levels at different times (3–24 h) was studied. Figure 4B shows a significant increase of GSH quantity after 12 and 24 h of treatment, suggesting that the effects of these compounds could likely be ascribed to their ability to activate the transcription of GSH.

### Activation of the Nrf2/ARE Pathway

An impairment of the transcriptional activity of Nrf2, the master regulator of neuronal adaptation to an oxidant environment, could be a consequence of an aberrant activity of the enzyme GSK-3β, which, in turn, is induced by pathological conditions including oxidative stress and disrupted redox balance. These events trigger a feed-forward loop, a distinct feature of neurodegenerative disorders, determining a decrease in cytoprotective effects against cellular insults. Recent studies indicate that GSK-3β, as a negative regulator of Nrf2, participates in the distribution of Nrf2 inside and outside the nucleus.^7^ In particular, the activation of GSK-3β leads to Nrf2 nuclear export and degradation at the cytosolic level.

**Figure 5:**
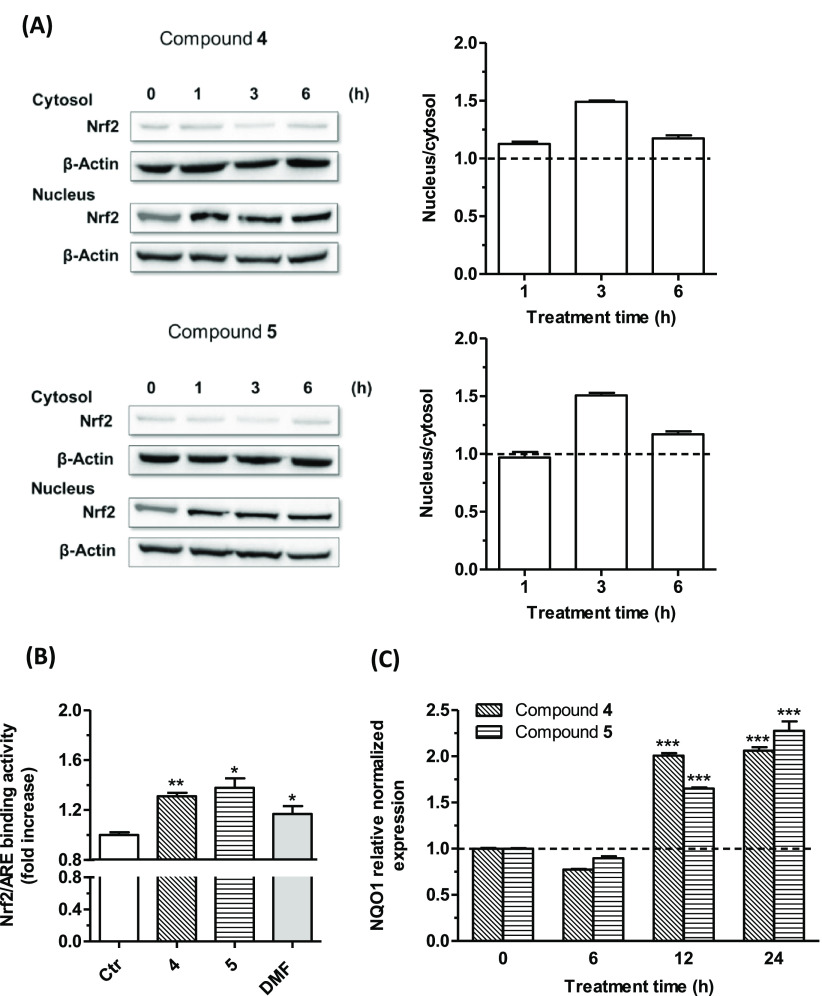
Effects of compounds **4** and **5** on the Nrf2/ARE signaling pathway (A and B) and NQO1 gene expression (C) in SH-SY5Y cells. (A) Cells were treated with compounds **4** and **5** (5 μM) for different times (1, 3, and 6 h). Translocation of Nrf2 from the cytosol to the nucleus was evaluated by Western blotting. Data are expressed as a ratio between nuclear Nrf2 and cytoplasmic Nrf2 levels and reported as mean ± SEM of at least three independent experiments. (B) Cells were treated with compounds **4** and **5** (5 μM) and DMF (5 μM) for 6 h. The Nrf2/ARE binding activity was determined by an ELISA assay. Results are expressed as fold increase versus untreated cells and reported as mean ± SEM of at least three independent experiments (** *p* < 0.01 and * *p* < 0.05 versus untreated cells at the *t*-test). (C) Cells were treated with compounds **4** and **5** (5 μM) for different times (6, 12, and 24 h). The NQO1 expression was determined by RT-PCR. Results are expressed as the relative normalized expression and reported as mean ± SEM of at least three independent experiments (*** *p* < 0.001 versus untreated cells at one-way ANOVA with the Dunnett post hoc test).

In light of these findings, **4** and **5** emerged as the most promising to be further studied regarding their effect on the Nrf2/ARE pathway. First, the nuclear-cytoplasmic Nrf2 shuttling cycle was studied in SH-SY5Y cells, and by observing the Western blotting, both tested compounds at 5 μM were seen to induce the nuclear translocation of Nrf2 both after short-term (1 and 3 h) and long-term treatments (6 h). The maximum ratio between nuclear and cytosolic Nrf2 levels was detected after a 3 h treatment for both compounds, while a slight reduction was observed after 6 h. These data suggested that compounds **4** and **5** induced a partial cytosolic redistribution of Nrf2 without altering its clearance (Figure 5A). Moreover, these results suggested a sustained Nrf2 activation together with a binding to the ARE sequence by the tested compounds. The activation and nuclear translocation of Nrf2 by **4** and **5** were also supported by an observed increase in Nrf2/ARE binding activity (Figure 5B). Briefly, these analogues showed superior effects to DMF, when tested at 5 μM.

To further confirm the increase in Nrf2 transcriptional activity upon treatment with **4** and **5**, the mRNA levels of NQO1, a Nrf2 target gene, were evaluated in SH-SY5Y cells (Figure 5C). In the experimental conditions, 5 μM of the tested compounds showed a significant ability to increase NQO1 mRNA levels in SH-SY5Y cells after 12 and 24 h of treatment.

### Neuroprotective Profile

The neuroprotective effects of curcumin-DEF hybrids **4** and **5** were investigated in some well-characterized *in vitro* models of AD and PD to gain insight into their therapeutic potential. In detail, the ability of the selected compounds to prevent SH-SY5Y cell death, induced by Aβ_1–42_ oligomers and 6-OHDA, was examined.

### In Vitro Model of AD

**Figure 6:**
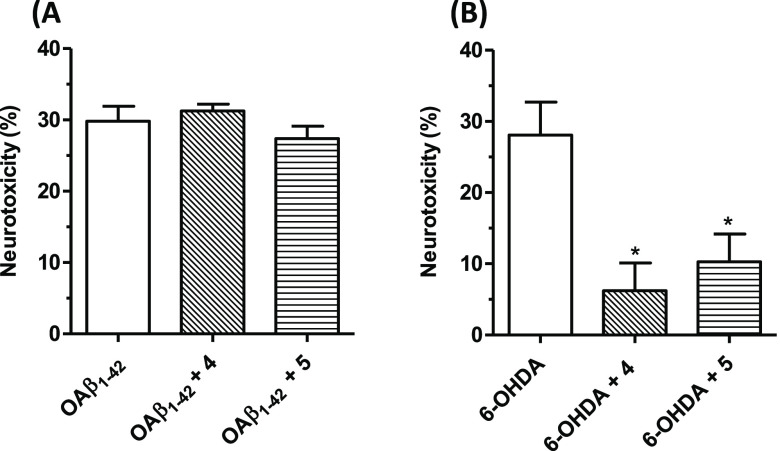
Effects of **4** and **5** on neurotoxicity induced by OAβ_1–42_ oligomers and 6-OHDA in SH-SY5Y cells. (A) Cells were incubated with **4** and **5** (5 μM, for 24 h) and then treated with OAβ_1–42_ (10 μM, for 4 h). (B) Cells were incubated with compounds **4** and **5** (5 μM, for 24 h), then treated with 6-OHDA (100 μM, for 2 h), and then starved in complete medium for 22 h. The neurotoxicity was evaluated by an MTT assay. Data are expressed as percentages of neurotoxicity versus cells treated with Aβ_1–42_ oligomers or 6-OHDA and reported as mean ± SEM of at least three independent experiments (* *p* < 0.05 versus cells treated with 6-OHDA at one-way ANOVA with the Dunnett post hoc test).

Intracellular accumulation of soluble Aβ_1–42_ oligomers is responsible for neurotoxic effects, such as neuronal death, and represents a key event in AD pathogenesis.^33^ Based on the promising data obtained, the most active compounds **4** and **5** were selected to be studied by using neurotoxic Aβ_1–42_ oligomers–based on an *in vitro* model of AD. Briefly, SH-SY5Y cells were incubated with 5 μM concentration of the selected analogues for 24 h, followed by OAβ_1–42_ oligomers treatment (10 μM) for 4 h (Figure 6A). Both tested compounds failed to prevent Aβ_1–42_ oligomers-induced cell death, indicative of their incapability to serve as Aβ-based therapeutics.

### In Vitro Model of PD

The neurotoxin 6-OHDA provides an *in vitro* lesion model by inducing CNS oxidative damage and neuroinflammation suitable for assessing the neuroprotective potential of PD therapeutics.^34^ Consistent with these effects, 6-OHDA (100 μM, for 2 h) was employed to induce damage in SH-SY5Y cells previously treated with **4** and **5** (5 μM, for 24 h). The results, depicted in Figure 6B, show that both the compounds mitigate the 6-OHDA-induced decrease in cell viability; this specific effect underlines potential usefulness of the tested analogues in the PD therapeutic area.

**Figure 7:**
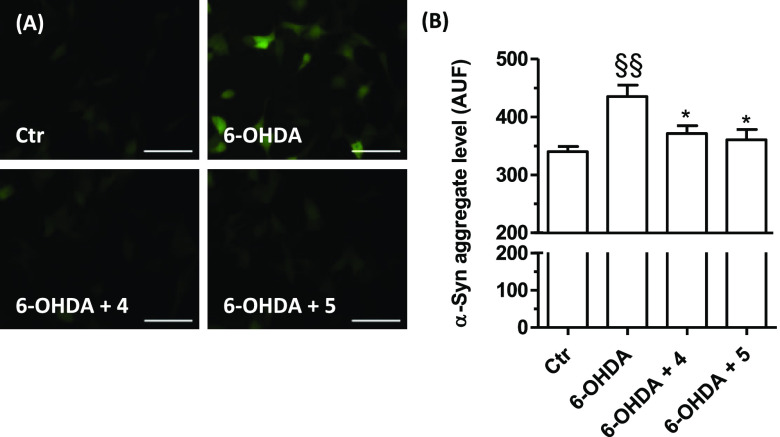
Effects of compounds **4** and **5** on α-syn aggregates induced by 6-OHDA in TagGFP2-α-syn SH-SY5Y cells. Cells were treated with compounds **4** and **5** (5 μM, for 24 h) and then with 6-OHDA (100 μM, for 2 h). At the end of incubation, the α-syn aggregates level was detected by fluorescence microscope. (A) Representative images of α-syn aggregates. (B) Quantification of the α-syn aggregates level. Data are expressed as mean fluorescence intensity ± SEM of at least three independent experiments (§§ *p* < 0.01 vs untreated cells, * *p* < 0.05 vs cells treated with 6-OHDA at one-way ANOVA with the Bonferroni post hoc test). Scale bars: 50 μm.

Assessment of the α-syn aggregate formation was performed to corroborate the above results. TagGFP2-α-syn SH-SY5Y cells were treated for 24 h with 5 μM of **4** and **5** and then with 100 μM of 6-OHDA. After a 2 h incubation, the presence of α-syn aggregates was visualized by fluorescence microscopy analysis and quantified (Figure 7). Both tested compounds were seen to significantly decrease the levels of toxic α-syn aggregates elicited by 6-OHDA.

### Neuroprotective Effects in an *C. elegans* Model of PD

**Figure 8:**
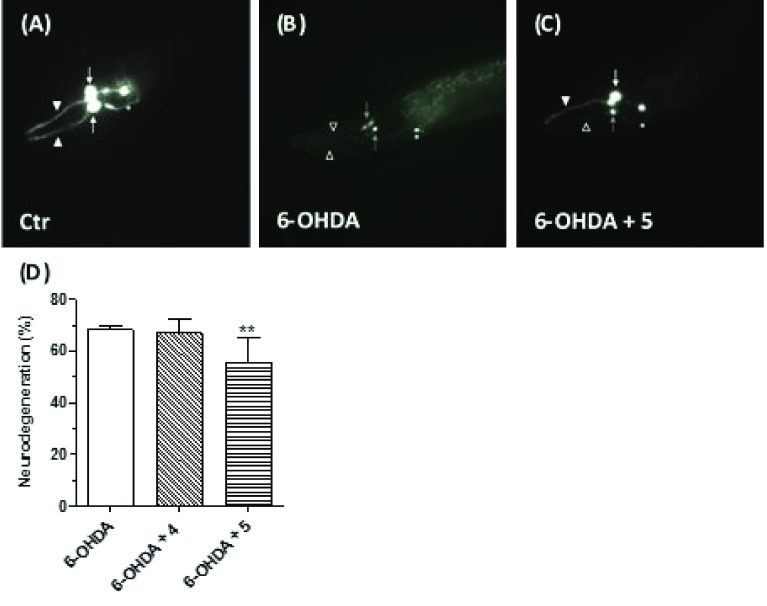
Effects of **4** and **5** on 6-OHDA-induced neurodegeneration in *C. elegans*. Animals were treated with **4** or **5** (5 μM) in the presence of 6-OHDA (5 mM) for 30 min. At the end of incubation, treated animals were placed on fresh agar plates for 72 h and then visualized as described in the Materials and Methods section. (A) In nontreated *vtIs7 [*p*dat-1::GFP]* transgenic animals, dopaminergic neurons express GFP, with two of the four CEP cell bodies (white arrows) and relative dendrites (arrowheads) visible in this focal plane in the head. (B) 6-OHDA treatment causes the degeneration of CEP dendrites (empty arrowheads) and two cell bodies (gray arrows), in treated animals. The other two CEP neurons are not visible anymore. (C) Compound **5** cotreatment partially rescues the 6-OHDA-induced toxic effects, with degeneration of one of the CEP dendrites (empty arrowhead) but not the other (arrowhead) and with one CEP cell body still viable (white arrow) and one dying (gray arrow) in this focal plane. Pictures have been taken with epifluorescence microscopy; in all panels, the anterior part of the animal is on the left and ventral down. ADE neurons, which are less affected by 6-OHDA treatment, are also visible but were not scored (asterisks). (D) Quantification of degenerating CEP neurons. Data are expressed as percentages of degenerating neurons and reported as mean ± SEM of at least three independent experiments. The number of animals observed is *n* = 270, 130, and 272, respectively (** *p* < 0.01 versus animals treated with 6-OHDA at one-way ANOVA with the Kruskal–Wallis post hoc test).

The nematode *Caenorhabditis elegans* (*C. elegans*) has been increasingly employed as a genetically tractable *in vivo* model to study the molecular mechanisms involved in several NDDs due to a short life cycle, fast reproduction, and a highly conserved inheritance (genetic basis).^35^ This invertebrate model offers the opportunity to take advantage of the significant conservation of genes and metabolic pathways common to both invertebrates and humans. At the neuronal level, the adult wild-type *C. elegans* organism contains only eight DA neurons that repeat the features of DA neurons of mammalian, making it possible to simplify the investigations considerably. In the transgenic *BY250 [vtIs7 (Pdat-1:GFP)]* strain, six DA neurons in the head, namely the four sensory cephalic (CEP) and the two anterior deirid (ADE) neurons, express the green fluorescent protein (GFP) and can be clearly visualized by fluorescence microscopy analysis and thus scored over time.^36^ Indeed, in this model, investigations of DA neurons degeneration *in vivo* are feasible through a first exposition of the nematode to PD-inducing toxin, namely 6-OHDA, followed by direct visualization of the GFP-labeled DA neurons.^37^ It is thus possible to screen small molecules for their anti-PD potential, in terms of rescue of the DA neurodegeneration phenotype manifested upon neurotoxin exposure, through visualization and scoring of the alterations of CEP neuron count or neuronal morphology. Interestingly, it has been previously demonstrated that in *C. elegans*, GSK-3, the orthologue of mammalian GSK-3α/β, prevents SKN-1 (orthologue of mammalian Nrf2) accumulation in the nuclei and constitutive induction of antioxidant gene expression in the intestine. This effect makes it possible the identification of neuroprotective agents with the ability to activate Nrf2 through the inhibition of GSK-3.^38^ The curcumin-DEF hybrids **4** and **5** were investigated in the above *C. elegans* model for their protective effects on 6-OHDA-induced degeneration of a subclass of DA neurons. Animals were incubated with 5 μM concentration of **4** and **5**, in the presence of 5 mM of 6-OHDA, for 30 min. After 72 h, the animals were studied by examining the four CEP neurons. Degenerating CEP neurons were morphologically analyzed and quantified (Figure 8). We recorded that in animals treated with 6-OHDA, 68% of CEP neurons degenerated. In contrast, cotreatment with **5** caused a partial rescue of this phenotype, with a decreased degeneration percentage (55%) of CEP neurons (Figure 8D). Otherwise, **4** cotreatment displayed no rescue of the phenotype (67% of neurons degenerating, Figure 8D). The visual study of DA neuronal cell bodies and neurites (Figures 8A–C) showed that in wild type untreated animals all CEP neurons are visible and show a normal morphology (Figure 8A), in animals treated with 6-OHDA dendrites and cell bodies degenerated (Figure 8B). The cotreatment with compound **5** prevented the degeneration of some of the CEP dendrites, and some CEP cell bodies were still visible, confirming a partial rescue of the 6-OHDA-induced toxic effects. From these results, it emerged that analogue **5**, but not **4**, can protect neurons from PD-inducing toxin insult *in vivo*.

Concerning the mechanisms that activate Nrf2 after treatment with the compounds **4** and **5**, our results suggest that part of the neuroprotective effects is Keap1-dependent. It is plausible that Nrf2 is released from Keap1 upon redox modifications and adduct formation with the molecules. However, other pathways, such as the activation of PI3K/AKT and subsequent inhibition of GSK-3β, a negative regulator of Nrf2, can also activate Nrf2. In this regard, under chronic oxidative stress observed in NDDs, the activation of GSK-3β can become predominant with an increase of the cytoplasmic degradation of Nrf2. Among the tested compounds, only **5** effectively inhibited GSK-3β activity, also showing neuroprotective effects in a transgenic *C. elegans* model of PD, where the activation of GSK-3β is closely involved in Nrf2 regulation as well as oxidative stress defense.^35^ The ability of the most potent compound **5** in a *C. elegans* model of PD to inhibit the GSK-3β kinase activation, in terms of inactive phospho-GSK-3α/β (Ser21/9) increase, was also evaluated in SH-SY5Y cells. After 1 h of incubation, compound **5** was seen to increase phospho-GSK-3α/β (Ser21/9) levels, suggesting the ability of this new derivative to inhibit the activation of GSK-3α/β also in neuronal cells (see Figure S1, Supporting Information).

#### Blood-Brain Barrier (BBB) Permeation

The BBB permeation represents an essential requirement for neurotherapeutic agents as it controls the capability of the drug to reach the CNS compartment at therapeutic concentrations. Thus, yet at an early drug discovery phase, the BBB permeability behavior of potential neurotherapeutics should be taken into account. This barrier is a compact boundary that manages exchanges between the CNS and the blood compartments.^39^ A parallel artificial membrane permeability assay (PAMPA) is a high-throughput technique developed to simulate the transcellular passive diffusion mechanism by which drugs reach the CNS. Thus, the capacity of all the newly synthesized curcumin-fumarate hybrids to enter the CNS was preliminarily explored using a PAMPA-BBB methodology employing a brain lipid porcine membrane.^40^ The *in vitro* permeability (*Pe*) of compounds **4**–**7** (Table 2) together with 10 commercial drugs (see Table S1, Supporting Information), through a lipid membrane extract, was determined. A good correlation between experimental-described values was obtained (see Figure S8, Supporting Information). Taking into account guidelines established in the literature for the prediction of BBB permeation, compounds showing a *Pe* value superior to 2.26 × 10^–6^ cm s^–1^ could be regarded as able to permeate the brain compartment and thus classified as (CNS +).^41^ Based on these premises, in light of the obtained results, all the synthesized curcumin-DEF hybrids (**4**–**6**) proved to be able to cross the BBB by passive permeation, while curcumin-fumaric acid (**7**) presented a borderline profile (CNS ±).

**Table 2:** Permeability (*Pe* 10^–6^ cm s^–1^) in the PAMPA-BBB Assay for Compounds **4**–**7** with Their Predictive Penetration in the CNS^a^

compound	*Pe* (10^–6^ cm s^–1^)^b^	prediction
4	2.5 ± 0.3	CNS +
5	4.8 ± 0.4	CNS +
6	4.5 ± 0.6	CNS +
7	1.7 ± 01	CNS +/CNS –

a The PBS:EtOH (70:30) mixture was used as solvent.

b Data are the mean ± SD of two independent experiments.

#### Chemical Stability Study

Conscious of the liabilities that might affect the newly synthesized curcumin-fumarate hybrids involving both the curcumin scaffold and the ester function, the chemical stability of the ester-based **6** and its carboxylic-acid counterpart **7** was studied by RP-HPLC.^21b^ In detail, by following a previously reported methodology, in the working conditions, compound **6** proved to be stable, while for derivative **7** the formation of a degradation product was observed (see Figure S10, Supporting Information).

## Conclusions

The significant therapeutic potential of multitarget agents for contrasting the multifactorial nature of NDDs prompted us to rationally design a new series of dual GSK-3β inhibitors/Nrf2 inducers. In detail, a rational design strategy was performed, by which previously discovered curcumin-based GSK-3β inhibitors (**1**–**3**) were joined to a fragment of a Nrf2 modulator, namely DEF. The obtained small series of curcumin-DEF hybrids (**4**–**7**) was tested against the selected targets GSK-3β and Nrf2, correlated to inflammation and oxidative stress, the well-recognized NDDs copathogenic factors. Regarding GSK-3β inhibition, derivatives **5** and **6** turned out to be the most effective of the series, showing low-micromolar potencies. In parallel, analogues **4** and **5** increased GSH intracellular levels through the activation of the Nrf2/ARE pathway, with particular reference to the capability to induce Nrf2 nuclear translocation and intensify Nrf2/ARE binding activity. These last derivatives emerged as dual GSK-3β/Nrf2 modulators and, due to their good BBB-penetrating capabilities, could be regarded as lead compounds, worth investigating for their neuroprotective potential. In detail, **4** and **5** demonstrated a protective effect against the neurotoxicity induced by 6-OHDA, which proved to be ineffective in protecting from Aβ_1–42_ oligomers insult. When studied in a transgenic *C. elegans* model of PD, compound **5** recorded a remarkable neuroprotective effect as, by observing transgenic *C. elegans* CEP dendrites and CEP cell bodies, its cotreatment provided a partial rescue of the toxic effects induced by 6-OHDA. Taken together, these data indicate derivative **5** as a very good lead compound for the development of PD therapeutics. Moreover, this molecule, due to its multipotent profile, could represent a lead compound worthy of further development to obtain disease-modifying PD therapeutics. In summary, this study corroborates the pivotal role of curcumin to serve as starting backbone for the design of multipotent therapeutics for NDDs cure.

## Materials and Methods

### Chemistry

#### General Procedures

Chemical reagents and solvents were employed as commercial products with a high-grade purity. Melting points were determined in open glass capillaries, using a Büchi apparatus, and are uncorrected. Reaction courses were monitored by thin-layer chromatography (TLC) performed on precoated TLC plates (Merck Silica Gel 60 F254, layer 0.20 mm) and then visualized under a UV lamp (λ = 254 and 365 nm). Flash column chromatography (FCC) separations were performed on silica gel (Kiesegel 40, particle size 0.040–0.063 mm, Merck). ^1^H NMR and ^13^C NMR spectra were recorded on a Varian INOVA spectrometer operating at 400 MHz; chemical shifts are reported as parts per million (ppm δ value). Standard abbreviations indicating spin multiplicities are given as follows: s (singlet), d (doublet), dd (doublet of doublet), t (triplet), br (broad), q (quartet), or m (multiplet). ESI-MS mass spectra were recorded on a Waters ZQ 4000 apparatus. All tested compounds were found to have >95% purity, as determined by RP-HPLC analysis, performed on a chromatograph MD210 plus diode array detector equipped with a 20 μL loop valve and a Pu 1580 pump (Jasco Europe, Italy) by using a Phenomenex Luna 5 μm C18 column (150 × 4.60 mm) as stationary phase, and a mixture of H_2_O/ACN/ (50:50 v/v) for **4** and **6** and H_2_O/ACN (40:60, v/v) for **5** and **7** as mobile phase; detection at λ = 280 and 425 nm, flow rate of 1.0 mL/min. Compound names are in accord with the naming algorithm developed by CambridgeSoft Corporation used in Chem-BioDraw Ultra 19.0. Compounds **1**–**3** have been synthesized as previously reported, and the spectroscopic data are in agreement with those reported in the literature.^21b^

#### General Procedure for the Synthesis of Curcumin-DEF Hybrids (**4**–**6**)

In a two neck flask equipped with a *nitrogen* inlet system and a rubber sept, NaH (60% dispersion in mineral oil, 1.5 molar equiv) was suspended in anhydrous THF (20.0 mL) and then dropwise added by a solution of **1**–**3** (1.00 mmol) in anhydrous THF (25.0 mL) at 0 °C; the obtained mixture was stirred for 30 min at 0 °C and then for 1 h at rt. Afterward, upon cooling to 0 °C, a solution of ethyl acetylenecarboxylate (2.0 molar equiv) in anhydrous THF (1.0 mL) was added *via* syringe, and the reaction was stirred at rt for 18 h. Then, the mixture was poured into ice/water (400 mL) followed by extraction with EtOAc (3 × 50.0 mL). The organic phases were combined and concentrated to dryness to give a residue that was then subjected to an FCC purification, followed by crystallization from a suitable solvent to afford the desired compounds **4**–**6**.

#### Ethyl (2*E*,4*Z*,6*E*)-5-Hydroxy-7-(4-methoxyphenyl)-4-((*E*)-3-(4-methoxyphenyl)acryloyl)hepta-2,4,6-trienoate (**4**)

According to the general procedure, reaction of NaH (0.09 g, 2.25 mmol), **1** (0.50 g, 1.5 mmol), and ethyl acetylenecarboxylate (0.30 mL, 3.0 mmol) gave a crude product that underwent FCC separation by using a 9.5:0.5 mixture of PE/EtOAc as eluent, followed by crystallization from EtOH, to obtain a red solid; mp 122–124 °C, 71% yield. ^1^H NMR (CDCl_3_): δ 1.37 (t, 3H, *J* = 7.2 Hz, CH_3_), 3.87 (s, 6H, OCH_3_), 4.31 (q, 2H, *J* = 6.8 Hz, OCH_2_), 5.96 (d, 1H, *J* = 15.6 Hz, =CH), 6.94 (d, 4H, *J* = 8.4 Hz, Ar), 7.00 (d, 2H, *J* = 15.6 Hz, =CH), 7.55 (d, 4H, *J* = 8.4 Hz, Ar), 7.79 (d, 2H, *J* = 15.2 Hz, =CH), 7.89 (d, 1H, *J* = 15.6 Hz, =CH) (see Figure S2, Supporting Information). ^13^C NMR (CDCl_3_): δ 14.5, 55.6 (2C), 60.7, 110.1, 114.6 (4C), 118.7 (2C), 122.7, 128.0 (2C), 130.4 (4C), 139.1, 142.7 (2C), 161.8 (2C), 167.0, 183.9 (2C) (see Figure S3, Supporting Information). MS-ESI (*m*/*z*): 457 (M + Na).

#### Ethyl (2*E*,4*Z*,6*E*)-7-(4-(Benzyloxy)phenyl)-5-hydroxy-4-((*E*)-3-(4-methoxyphenyl)acryloyl)hepta-2,4,6-trienoate (**5**)

According to the general procedure, reaction of NaH (0.09 g, 2.25 mmol), **2** (0.40 g, 1.00 mmol), and ethyl acetylenecarboxylate (0.30 mL, 3.0 mmol) gave a crude product that underwent FCC separation by using a 9.5:0.5 mixture of PE/EtOAc as eluent, followed by two sequential treatments with EtOAc/nE and EtOH. A dark red solid was obtained, mp 63–65 °C, 41% yield. ^1^H NMR (CDCl_3_): δ 1.35 (t, 3H, *J* = 7.2 Hz, CH_3_), 3.87 (s, 3H, OCH_3_), 4.31 (q, 2H, *J* = 6.8 Hz, OCH_2_), 5.13 (s, 2H, Bn OCH_2_), 5.96 (d, 1H, *J* = 15.6 Hz, =CH), 6.94 (d, 2H, *J* = 8.7 Hz, Ar), 7.00 (d, 2H, *J* = 15.2 Hz, =CH), 7.01 (d, 2H, *J* = 8.0 Hz, Ar), 7.32–7.47 (m, 5H, Bn Ar), 7.55 (d, 4H, *J* = 7.6 Hz, 4CH, Ar), 7.78 (d, 2H, *J* = 15.6 Hz, CH=CH), 7.89 (d, 1H, *J* = 15.6 Hz, CH=CH) (see Figure S4, Supporting Information). ^13^C NMR (CDCl_3_, 101 MHz): δ 14.5, 55.6, 60.7, 70.3, 110.1, 114.6 (2C), 115.5 (2C), 118.7, 118.9, 122.7, 127.6 (2C), 127.9, 128.2, 128.3, 128.8 (2C), 130.4 (4C), 136.5, 139.1, 142.6, 142.7, 161.0, 161.8, 167.0, 183.8 (2C) (see Figure S5, Supporting Information). MS-ESI (*m*/*z*): 533 (M + Na).

#### Ethyl (2*E*,4*Z*,6*E*)-7-(4-(Benzyloxy)phenyl)-4-((*E*)-3-(4-(benzyloxy)phenyl)acryloyl)-5-hydroxyhepta-2,4,6-trienoate (**6**)

According to the general procedure, reaction of NaH (0.09 g, 2.25 mmol), **3** (0.50 g, 1.00 mmol), and ethyl acetylenecarboxylate (0.30 mL, 3.0 mmol) gave a crude product that underwent FCC separation by using a 9.75:0.25 mixture of PE/acetone as eluent, followed by two sequential treatments with DCM/PE and EtOH. A yellow-brown solid was obtained, mp 153–155 °C, 84% yield. ^1^H NMR (CDCl_3_, 400 MHz): δ 1.38 (t, 3H, *J* = 7.2 Hz, CH_3_), 4.31 (q, 2H, *J* = 6.8 Hz, OCH_2_), 5.13 (s, 4H, Bn OCH_2_), 5.95 (d, 1H, *J* = 15.6 Hz, =CH), 6.98 (d, 4H, *J* = 8.8 Hz, Ar), 6.99 (d, 2H, *J* = 15.4 Hz, =CH), 7.32–7.45 (m, 11H, OH and Bn Ar), 7.55 (d, 4H, *J* = 8.8 Hz, Ar), 7.78 (d, 2H, *J* = 15.2 Hz, =CH), 7.88 (d, 1H, *J* = 15.6 Hz, =CH) (see Figure S6, Supporting Information). ^13^C NMR (CDCl_3_, 101 MHz): δ 14.5, 60.7, 70.3 (2C), 110.1, 115.5 (4C), 118.9 (2C), 122.7, 127.6 (4C), 128.2 (2C), 128.3 (2C), 128.8 (4C), 130.4 (4C), 136.5 (2C), 139.1, 142.6 (2C), 161.0 (2C), 167.0, 183.8 (2C) (see Figure S7, Supporting Information). MS-ESI (*m*/*z*): 609 (M + Na).

#### (2*E*,4*Z*,6*E*)-7-(4-(Benzyloxy)phenyl)-4-((*E*)-3-(4-(benzyloxy)phenyl)acryloyl)-5-hydroxyhepta-2,4,6-trienoic Acid (**7**)

A ΚOH methanol solution (2.0 N, 0.62 mL) was added dropwise to a solution of **6** (0.10 g, 0.17 mmol) in MeOH (4.4 mL) during a period of 30 min and under stirring; the obtained mixture was heated at 60 °C under stirring for 12 h. Then, the solvent was removed under reduced pressure to give a residue that was solubilized with Et_2_O (30 mL) and then extracted with H_2_O (3 × 30 mL). The aqueous phase was acidified with 37% HCl and then extracted with DCM (3 × 30 mL). The combined organic layers were washed with brine, dried over Na_2_SO_4_, filtered, and concentrated to dryness, affording a residue that was purified by two sequential treatments with DCM/PE and EtOH. A pale brown powder was obtained, mp 182–184 °C, 75% yield. ^1^H NMR (acetone-*d*_6_, 400 MHz): δ 5.19 (s, 4H, Bn OCH_2_), 6.39 (d, 2H, *J* = 15.6 Hz, =CH), 7.08 (d, 4H, *J* = 8.8 Hz, Ar), 7.34–7.42 (m, 10H, Bn Ar), 7.49 (d, 4H, *J* = 7.6 Hz, Ar), 7.63 (d, 4H, *J* = 16.4 Hz, =CH), 7.63 (br, 1H, OH). ^13^C NMR (acetone-*d*_6_, 101 MHz): δ 70.0 (2C), 110.1, 116.0 (4C), 119.0 (2C), 122.7, 127.6 (4C), 128.1 (2C), 128.3 (2C), 128.8 (4C), 130.5 (4C), 136.5 (2C), 139.0, 142.6 (2C), 160.9 (2C), 168.0, 183.9 (2C). ESI-MS-(*m*/*z*): 581 (M + Na).

#### GSK-3β Inhibition

The method of Baki et al. was followed for the inhibition of GSK-3β (see the Supporting Information for experimental details).^28^

#### Kinetic Studies

Kinetic experiments were performed using the ADP-Glo Kinase Assay,^42^ in which ATP concentration varied from 1 to 50 μM, the concentrations of inhibitors employed were 5 and 10 μM, while the concentration of GS2 was kept constant (25 μM). Graphs (Figure 2) report the double reciprocal plot of the data.

#### Cell Cultures

Human neuronal SH-SY5Y cells (Sigma-Aldrich, St. Louis, MO, USA) and TagGFP2-α-synuclein SH-SY5Y cells (Innoprot, Bizkaia, Spain) were routinely grown in Dulbecco’s modified Eagle’s Medium supplemented with 10% fetal bovine serum, 2 mM l-glutamine, 50 U/mL penicillin, and 50 μg/mL streptomycin at 37 °C in a humidified incubator with 5% CO_2_.

#### Intracellular ROS Formation

SH-SY5Y cells were seeded in a 96-well plate at 2 × 10^4^ cells/well, incubated for 24 h, and then treated with 5 μM concentration of compounds **4**–**7** and 5–10 μM concentrations of DMF at 37 °C for 24 h, in 5% CO_2_. ROS formation was evaluated by using the fluorescent probe H_2_DCF-DA, as previously described.^43^

#### Measurement of Intracellular GSH

SH-SY5Y cells were seeded in a black 96-well plate at 2 × 10^4^ cells/well, incubated for 24 h, and then treated with compounds **4**–**7** (5 μM) and DMF (5–10 μM) for 24 h at 37 °C in 5% CO_2_. In parallel, SH-SY5Y cells were seeded in 60 mm dishes at 2 × 10^6^ cells/dish, incubated for 24 h, and then treated with curcumin-DEF hybrids **4** and **5** (5 μM) for 3, 6, 12, and 24 h at 37 °C in 5% CO_2_. GSH levels were evaluated by using the fluorescent probe MCB, as previously described.^44^

#### Detection of Nrf2 Nuclear Translocation by Western Blotting

SH-SY5Y cells were seeded in 60 mm dishes at 2 × 10^6^ cells/dish, incubated for 24 h, and then treated with compounds **4** and **5** (5 μM) for 1, 3, and 6 h at 37 °C in 5% CO_2_. At the end of treatment, cytosolic and nuclear extractions for Nrf2 nuclear translocation were performed by using a Nuclear Extract Kit (Active Motif, Carlsbad, CA, USA), according to the manufacturer’s guidelines. Cytosolic and nuclear extracts (50 μg per sample) were separated by SDS-polyacrylamide gels and transferred onto nitrocellulose membranes, which were probed with primary Nrf2 (1:1000; Santa Cruz Biotechnology, Dallas, TX, USA) and secondary antibodies. ECL reagents (Pierce) were utilized to detect targeted bands. The same membranes were stripped and reprobed with β-actin antibody (1:1000; Sigma-Aldrich). Western blot bands were analyzed by densitometry, using Quantity One software (Bio-Rad, Hercules, CA, USA). Results are expressed as a ratio between nuclear and cytoplasmatic Nrf2 levels.

#### Determination of the Active Nrf2 Protein Level

The levels of active Nrf2 protein were determined on the nuclear extracts (10 μg, see above) by using the DNA-binding ELISA TransAM Nrf2 Kit (Active Motif), according to the manufacturer’s guidelines. In detail, the kit comprises a primary antibody able to recognize an epitope on Nrf2 protein upon ARE binding. In the treated cells, the amount of active Nrf2 protein is expressed as a fold increase in comparison to the corresponding untreated cells.

#### RNA Extraction and Quantitative Real-Time PCR

SH-SY5Y cells were seeded in 100 mm dishes at 2.5 × 10^6^ cells/dish, incubated for 24 h, and then treated with compounds **4** and **5** (5 μM) for different times (6, 12, and 24 h) at 37 °C and in 5% CO_2_. Afterward, a cell suspension was pelleted, and RNA was extracted by the PureLink RNA Mini Kit (Life Technologies, Carlsbad, CA, USA) according to the manufacturer’s guidelines. A total of 1 μg of RNA were used to synthesize cDNA using the SuperScript VILO MasterMix (Invitrogen, Carlsbad, CA, USA). Quantitative Real-Time PCR was carried out using SYBR Select Master Mix (Invitrogen), and relative normalized expression was calculated by comparing the cycle threshold (Ct) of the target gene to that of the reference genes beta-2 microglobulin (B2M) and TATA-box binding protein (TBP, Life Technologies). All reactions had three technical replicates, and each condition had three biological replicates. Relative quantification was calculated according to the ΔΔCt method (2^–ΔΔCt^) with untreated cells as control. Primer sequences used in this study are listed in Table S2.

#### Aβ_1–42_ Oligomers Preparation for the Determination of Neuroprotective Activity

Aβ_1–42_ peptide (AnaSpec, Fremont, CA, USA) was first dissolved in 1,1,1,3,3,3-hexafluoroisopropanol to 1 mg/mL, sonicated, incubated at rt for 24 h, and lyophilized to obtain an unaggregated Aβ_1–42_ peptide film that was solubilized with DMSO and stored at −20 °C until use. The aggregation of Aβ_1–42_ peptide into oligomers was performed as previously described.^45^

#### Neuroprotective Activity toward Aβ_1–42_ Oligomers

SH-SY5Y cells were seeded in a 96-well plate at 3 × 10^4^ cells/well, incubated for 24 h, and treated with compounds **4** and **5** (5 μM) for 24 h. Then, cells were treated with Aβ_1–42_ oligomers for 4 h. The neuroprotective activity, in terms of an increase in intracellular MTT granules, was measured by an MTT assay, as previously described (see the Supporting Information for experimental details).^46^

#### Neuroprotective Activity toward 6-Hydroxydopamine

SH-SY5Y cells were seeded in a 96-well plate at 3 × 10^4^ cells/well, incubated for 24 h, and subsequently treated with compounds **4** and **5** (5 μM) for 24 h. Then, cells were treated with 6-hydroxydopamine (6-OHDA, 100 μM) for 2 h and starved in complete medium for 22 h. The neuroprotective activity was measured by using the MTT assay as previously described.^47^ Data are expressed as a percentage of neurotoxicity versus untreated cells.

#### Detection of α-Synuclein Aggregation

TagGFP2-α-synuclein SH-SY5Y cells were seeded in a 96-well plate at 2 × 10^4^ cells/well, incubated for 24 h, and subsequently treated with curcumin-DEF hybrids **4** and **5** (5 μM) for 24 h at 37 °C in 5% CO_2_. Then, cells were treated with 6-OHDA (100 μM) for 2 h at 37 °C in 5% CO_2_. At the end of incubation, the aggregation of α-synuclein was detected using an inverted fluorescent microscope (Eclipse Ti-E, Nikon Instruments Spa, Florence, Italy). The intensity of fluorescence was directly proportional to the aggregation of α-synuclein. Data are expressed as arbitrary units of fluorescence.

#### *C. elegans* Methods

Nematodes were grown and handled following standard procedures, under uncrowded conditions, at 20 °C, on nematode growth medium (NGM) agar plates seeded with *Escherichia coli* strain OP50.^48^ The transgenic strain used in this work is BY250 *vtIs7[pdat-1::Green Fluorescent Protein (GFP)]*.^36^ 6-OHDA toxicity against DA neurons was assessed as previously described.^37a,49,37b^*vtIs7[pdat-1::GFP]]* were synchronized, and L1 larval stage animals were washed off the plate and incubated with a solution of 1% DMSO (Sigma-Aldrich), 1 mM ascorbic acid (Sigma-Aldrich), and 5 mM 6-OHDA (Sigma-Aldrich) for 0.5 h in dark conditions and gentle agitation (as control, to exclude that any rescue was caused by the DMSO solvent and not by synthetic compounds) or with a solution of compounds **4** or **5** (5 μM) dissolved in 1% DMSO, 1 mM ascorbic acid, and 5 mM 6-OHDA. Treated animals were transferred onto plates (without removal of 6-OHDA and the compounds), incubated for 72 h, and then placed on a freshly prepared 4% agar pad, immobilized using 0.01% tetramisole hydrochloride (Sigma-Aldrich). Animals were visualized using Zeiss Axioskop, equipped with epifluorescence and images collected with a Leica DM6000B microscope equipped with epifluorescence and the digital camera Hamamatsu C11440. Only the four dopaminergic CEP neurons were analyzed and scored as degenerated when we observed any of the following defects: blebbing, disappearance or breaking of the dendrites, and disappearance or rounding of the cell bodies. Results are expressed as a percentage of degenerating neurons.

#### Statistical Analysis

Results are shown as mean ± standard error (SEM) of three independent experiments. Statistical analysis was performed using one-way ANOVA with the Dunnett or Bonferroni or Kruskal–Wallis post hoc test and Student’s *t*-test, as appropriate. Differences were considered significant at *p* < 0.05. Analyses were performed using GraphPad PRISM software (version 5.0; GraphPad Software, La Jolla, CA, USA) on a Windows platform.

#### CNS Penetration

An *in vitro* parallel artificial membrane permeability assay (PAMPA)-blood-brain barrier (BBB) was employed. Prediction of the brain penetration was evaluated using a parallel artificial membrane permeability assay (PAMPA)^40^ (see the Supporting Information for experimental details).

#### Chemical Stability Study

The tested derivatives **6** and **7** were dissolved in DMSO (0.50 mg/mL), and the pH of the solution was adjusted to 7.4 using 50 mM phosphate. The obtained solutions were then maintained at 70 °C (oven) for 24 h and analyzed by RP-HPLC under the conditions reported in the General Procedures section.

## Supporting Information Available

NMR spectra of compounds **4**–**6**; GSK-3β activity of compound **5**; PAMPA-BBB assay: *Pe* values of 10 commercial drugs and linear correlation among experimental-reported *Pe*; cytotoxicity of compounds **4**–**7**, TDZD, and DMF in SH-SY5Y cells; primer sequences for quantitative real-time PCR; RP-HPLC chromatograms (compounds **6** and **7**); and Materials and Methods (PDF)

## Abbreviations

AD: Alzheimer’s disease
Aβ: amyloid-β
ADE: anterior deirid
ARE: antioxidant response element
BACE-1: β-site amyloid precursor protein cleaving enzyme
BBB: blood-brain barrier
CEP: cephalic
CNS: central nervous system
H_2_DCF-DA: 2′,7′-dichlorodihydrofluorescein diacetate
DEF: diethyl fumarate
DMF: dimethyl fumarate
MTT: 3-(4,5-dimethylthiazol-2-yl)-2,5-diphenyltetrazolium bromide
FCC: flash column chromatography
FAEs: fumaric acid esters
GSH: glutathione
GSK-3β: glycogen synthase kinase-3β
GFP: green fluorescent protein
Keap1: Kelch-like ECH associated protein1
LBs: Lewy bodies
6-OHDA: 6-hydroxydopamine
NQO1: NAD(P)H: quinone oxidoreductase 1
NDDs: neurodegenerative diseases
Nrf2: nuclear factor-erythroid related factor 2
NF-kB: nuclear factor-kB
MCB: monochlorobimane
PD: Parkinson’s disease
p-tau: phospho-tau
ROS: reactive oxygen species
α-syn: α-synuclein
*t*-BuOOH: *tert*-butyl hydroperoxide
TLC: thin-layer chromatography
