# Supplementary material for: Novel Curcumin-Diethyl Fumarate Hybrid as a Dualistic GSK-3β Inhibitor/Nrf2 Inducer for the Treatment of Parkinson’s Disease
Source: ACS Chem Neurosci. 2020 Jul 14;11(17):2728–40. doi: 10.1021/acschemneuro.0c00363 (PMC8009478; doi:10.1021/acschemneuro.0c00363)
Supplement: Supplementary file 1 — cn0c00363_si_001.pdf [file cn0c00363_si_001.pdf]

Supporting Information

Novel Curcumin-Diethyl Fumarate Hybrid as a Dualistic GSK-3β Inhibitor/Nrf2 Inducer for the Treatment of Parkinson's Disease

Rita Maria Concetta Di Martino[†§^], Letizia Pruccoli[‡^], Alessandra Bisi[†], Silvia Gobbi[†], Angela Rampa[†], Ana Martinez[ǁ], Concepción Pérez[ǁ], Loreto Martinez-Gonzalez[ǁ], Maria Paglione[#], Elia Di Schiavi[#], Francesca Seghetti[†], Andrea Tarozzi[‡]* and Federica Belluti[†]*

[†]Department of Pharmacy and Biotechnology, Alma Mater Studiorum - University of Bologna, Via Belmeloro 6, 40126, Bologna, Italy
[‡]Department for Life Quality Studies, Alma Mater Studiorum - University of Bologna, Corso d'Augusto 237, 47921, Rimini, Italy
[ǁ]Centro de Investigaciones Biologica, CSIC, Ramiro de Maeztu 9, 28040, Madrid, Spain
[#]Institute of Biosciences and BioResources (IBBR), Department of Biology, Agriculture and Food Science, National Research Council (CNR), Via Pietro Castellino 111, 80131, Naples, Italy
¥Centro de Investigación Biomédica en Red de Enfermedades Neurodegenerativas (CIBERNED), Instituto Carlos III, 28031 Madrid (Spain)
[§]Present address: Department of Drug Discovery and Development, Istituto Italiano di Tecnologia, via Morego 30, 16163, Genoa, Italy
^These authors contributed equally to this work
Correspondence to: federica.belluti@unibo.it

Contents

Figure S1. GSK-3β activity of compound **5.** S1

Figure S2. ^{1}H-NMR Spectrum of compound **4.** S1

Figure S3. ^{13}C-NMR Spectrum of compound **4**. S2

Figure S4. ^{1}H-NMR Spectrum of compound **5**. S2

Figure S5. ^{13}C-NMR Spectrum of compound **5**. S3

Figure S6. ^{1}H-NMR Spectrum of compound **6**. S3

Figure S7. ^{13}C-NMR Spectrum of compound **6**. S4

Table 1. *Pe* values of 10 commercial drugs (PAMPA-BBB assay). S4

Figure S8. Linear correlation among experimental-reported *Pe* (PAMPA-BBB assay). S5

Figure S9. Cytotoxicity of compounds **4-7**, TDZD, and DMF in SH-SY5Y cells. S5

Table S2. Primer sequences for quantitative Real-Time PCR. S5

Figure S10. Stability Study. RP-HPLC chromatograms (compounds **6** and **7**). S6

MATERIALS AND METHODS S6

GSK-3β inhibition. S6

Cell viability. S6

Detection of phosphorylated GSK-3β kinase by Western blotting. S6

BBB permeation. S7

References. S7

GSK-3β activity in SH-SY5Y cells

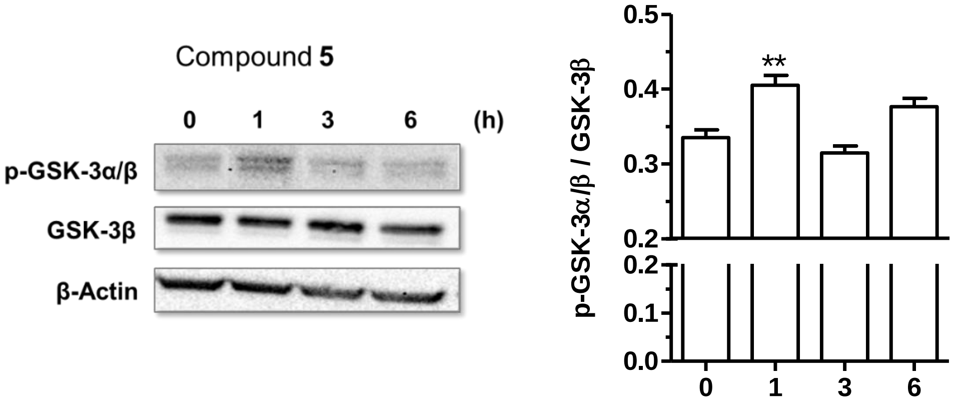

Figure S1. Inhibition of GSK-3β activity in SH-SY5Y cells. Cells were incubated with compound **5** (5 μM) for different times (1, 3 and 6 h). At the end of incubation, the phosphorylation of GSK-3α/β (Ser21/9) was determined by Western blotting, as described in the materials and methods section. Data are expressed as the ratio between phospho-GSK-3α/β and GSK-3β levels normalised against β-Actin and reported as mean ± SEM of at least three independent experiments (** $p < 0.01$ versus untreated cells at one-way ANOVA with Dunnett post hoc test).

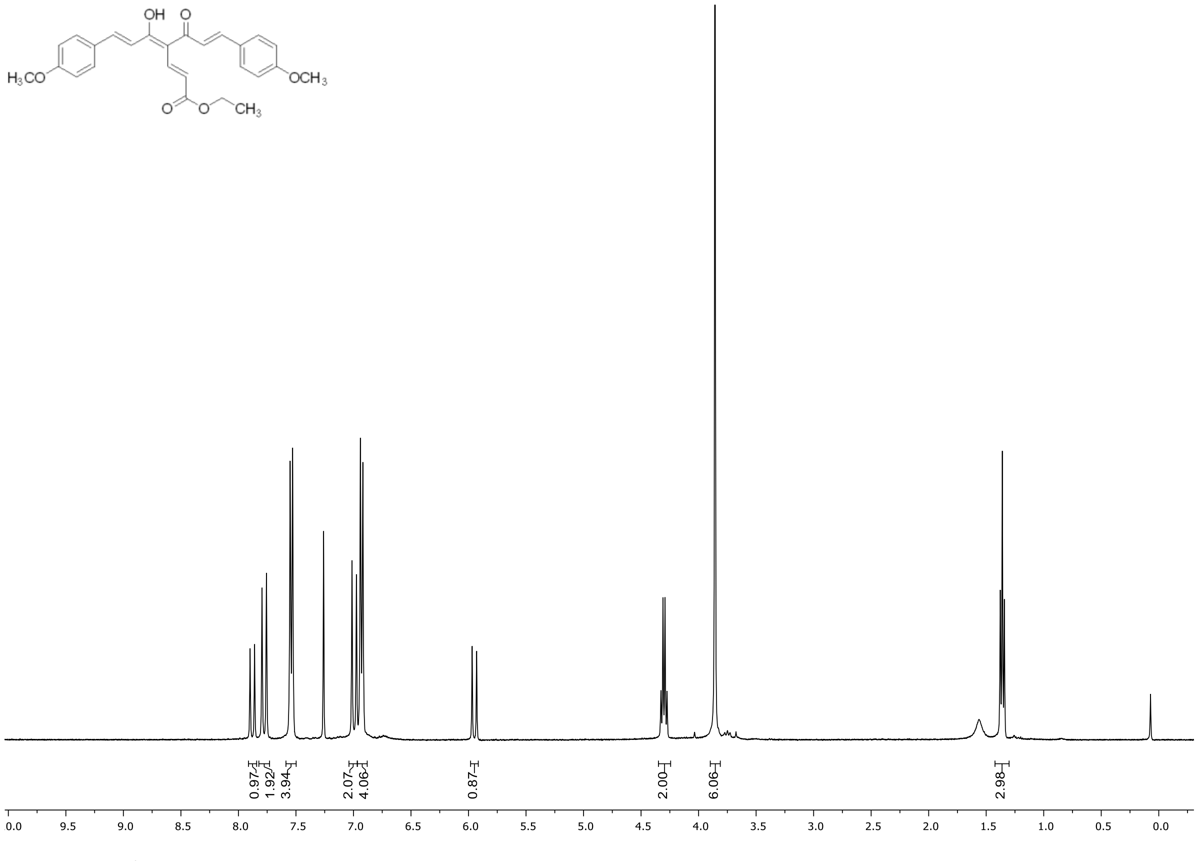

Figure S2. ^{1}H-NMR ($CDCl_3$, 400 MHz) of compound **4**.

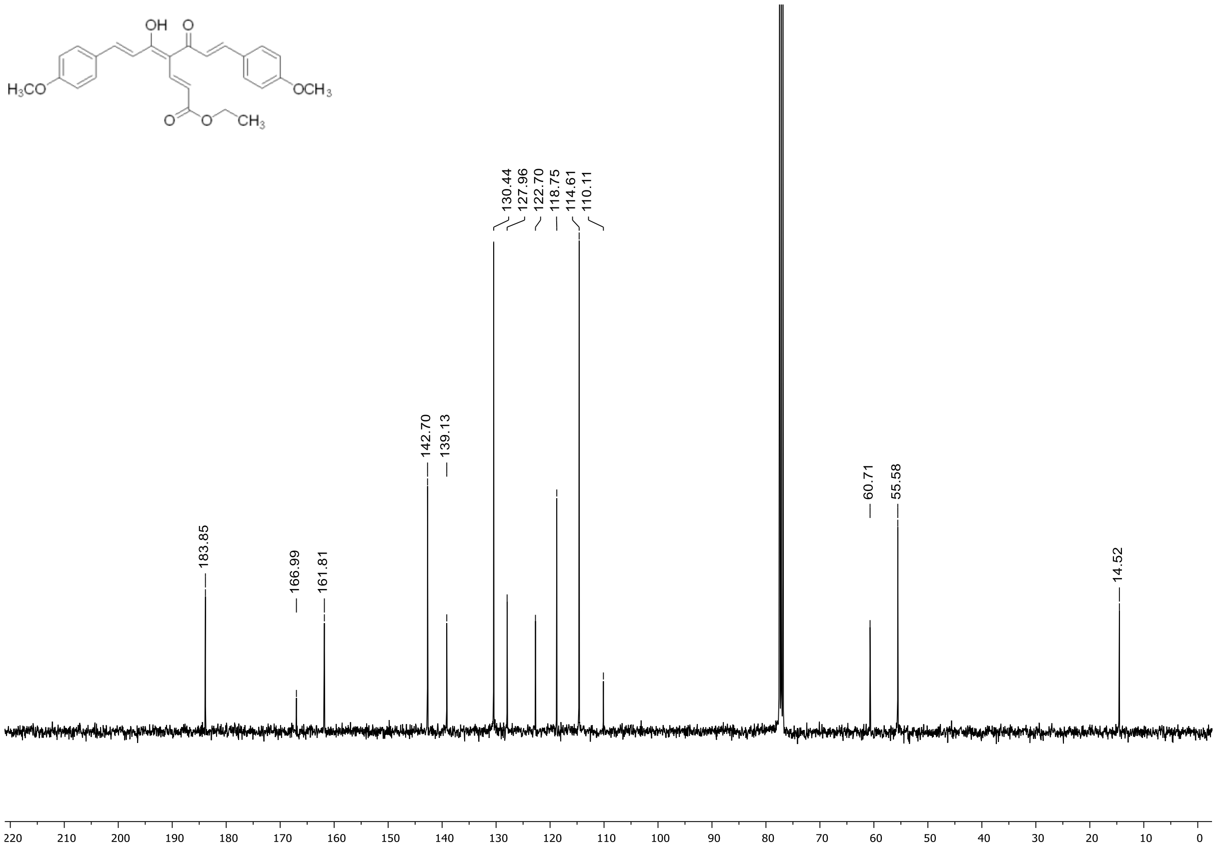

Figure S3. ^{13}C-NMR ($CDCl_3$, 101 MHz) of compound **4.**

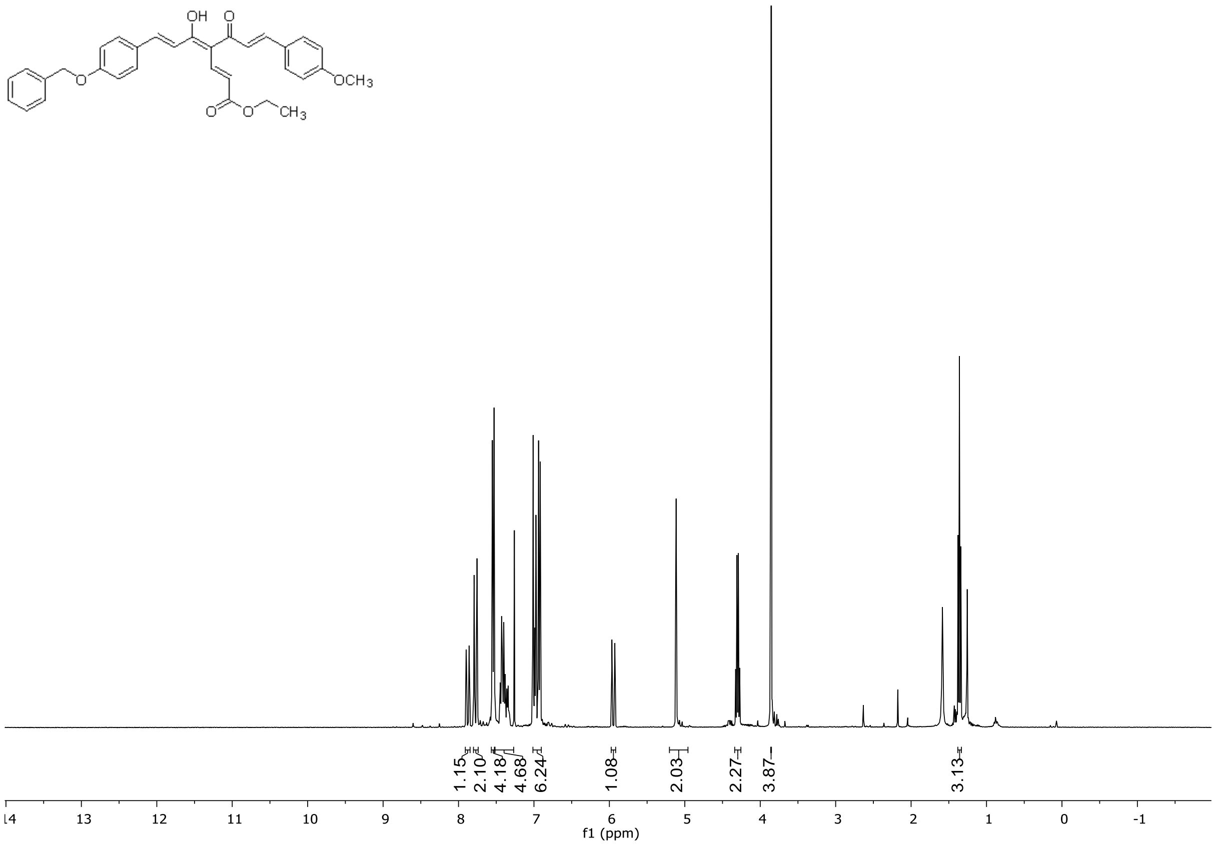

Figure S4. ^{1}H-NMR ($CDCl_3$, 400 MHz) of compound **5**.

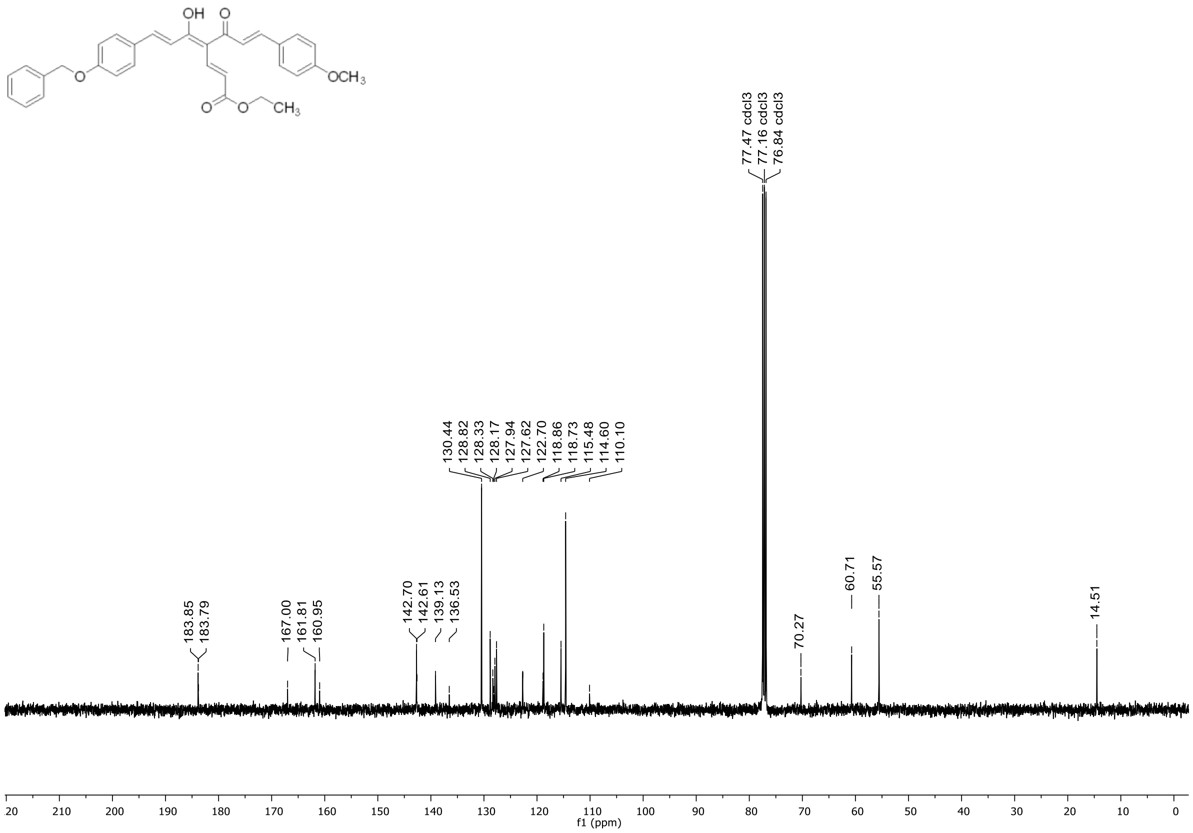

Figure S5. ^{13}C-NMR ($CDCl_3$, 101 MHz) of compound **5.**

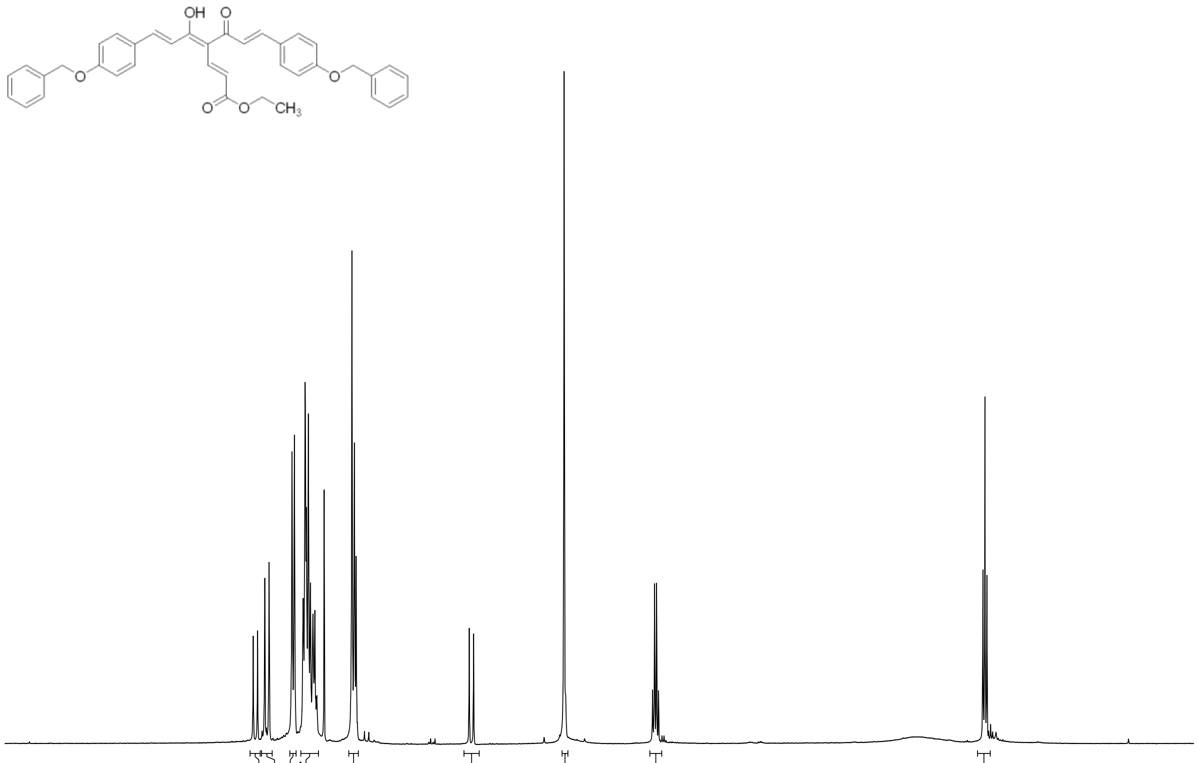

Figure S6. ^{1}H-NMR ($CDCl_3$, 400 MHz) of compound **6**.

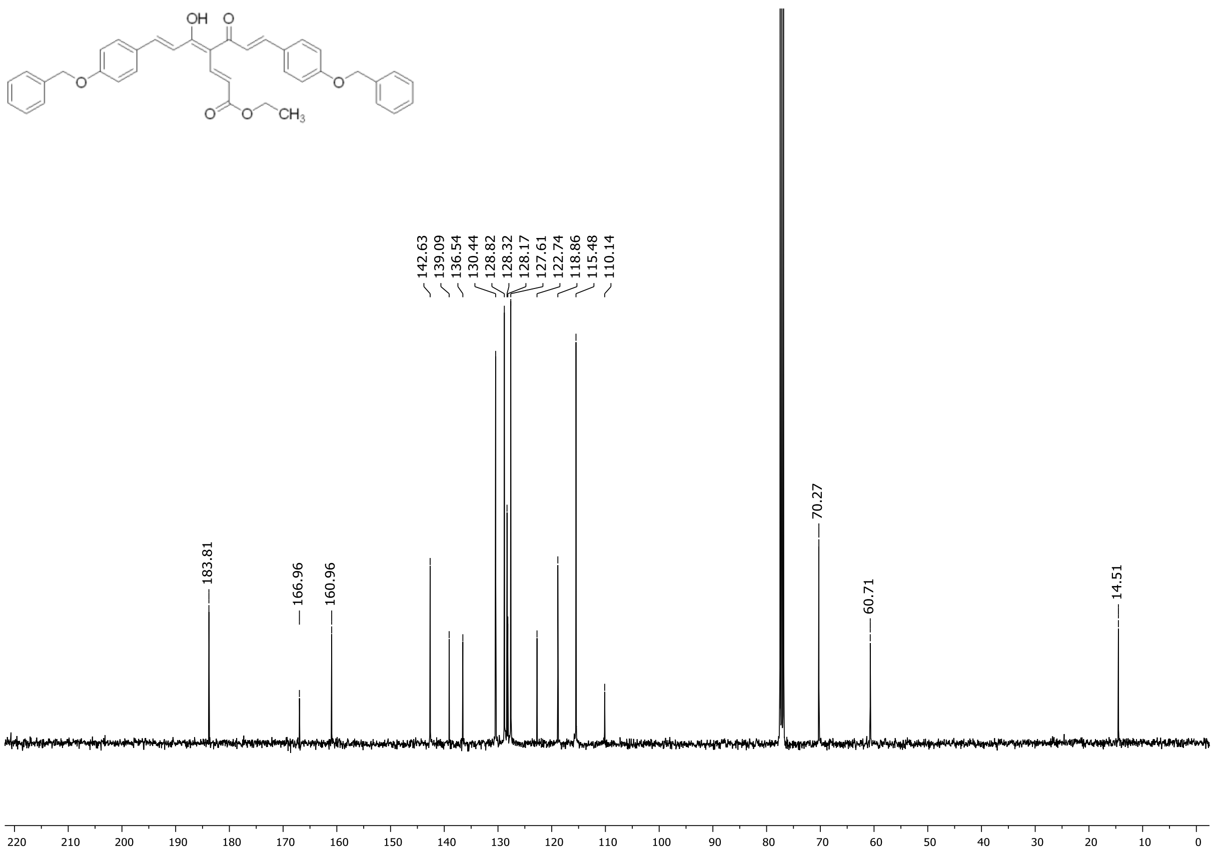

Figure S7. ^{13}C-NMR ($CDCl_3$, 101 MHz) of compound **6**.

Blood-Brain Barrier (BBB) Permeation

To experimentally validate the PAMPA-BBB assay, *in vitro* permeabilities (*Pe*) values of commercial drugs were determined (Table 1) and then compared with the experimental data of the new compounds, obtained employing this methodology.

Table S1. Permeability (*Pe* 10^{-6} cm s^{-1}) in the PAMPA-BBB assay for 10 commercial drugs (used in the experiment validation) with their predictive penetration in the CNS.[a]

Compound	Bibl.[b]	*Pe* (10^{-6} cm s^{-1})[c]		
Atenolol	0.8	0.3	±	0,1
Caffeine	1.3	0.5	±	0,1
Desipramine	12	8,7	±	1,0
Enoxacin	0.9	0.5	±	0,1
Hydrocortisone	1.9	0.8	±	0,6
Ofloxacin	0.8	0.2	±	0,1
Piroxicam	2.5	0.4	±	0,1
Promazine	8.8	5.9	±	0,7
Testosterone	17	10.0	±	1,1
Verapamil	16	9.8	±	0,5

[a]PBS:EtOH (70:30) was used as solvent. [b] See Reference [1]. [c]Datmonga are the mean ± SD of 2 independent experiments.

A good correlation (R^2= 0.9803) between experimental (exptl) and the corresponding values described in literature (bibl) was obtained: *Pe* (exptl) = 0.6507 (bibl) – 0.3341 (Figure S8).

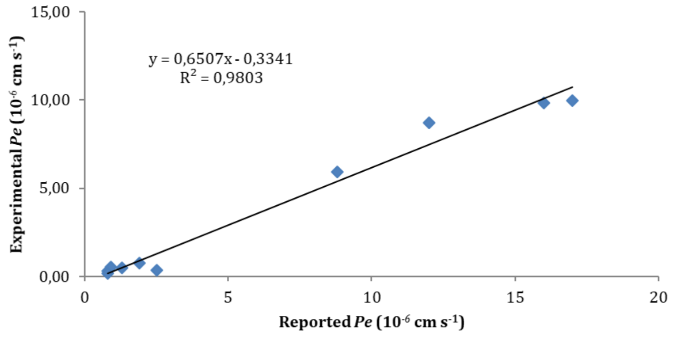

Figure S8. Linear correlation among experimental and reported permeability of commercial drugs using the PAMPA-BBB assay.

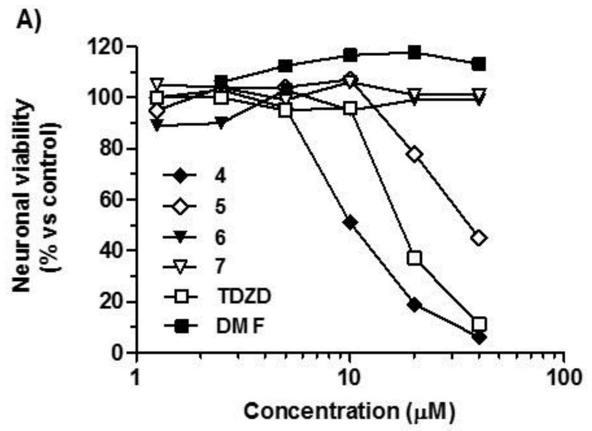

B)

Compound	IC_{50} (µM)[a]
4	18.55
5	30.71
6	> 40[b]
7	> 40[b]
TDZD	18.46
DMF	> 40[b]

[a]Concentration resulting in 50% inhibition of neuronal viability; [b]Maximum concentration used

Figure S9. Cytotoxicity of compounds 4-7, TDZD and DMF in SH-SY5Y cells. A) Cells were incubated for 24 h with different concentrations of the studied compounds [1.25 - 40 µM]. At the end of incubation, the neuronal viability was measured using MTT assay, as described in the materials and methods section. Data are expressed as percentage of neuronal viability versus untreated cells and reported as mean of two independent experiments; B) IC_{50} values of compounds.

Table S2. Primer sequences for quantitative Real-Time PCR.

Gene name	Forward/Reverse	5' to 3' Sequence
NQO1	For	GGGATCCACGGGGACATGA
	Rev	ATTTGAATTCGGGCGTCTGC
B2M	For	CTTTCCATTCTCTGCTGGATGACG
	Rev	GCGGGCATTCCTGAAGCTGACAGCA
TBP	For	CACATCACAGCTCCCCACCA
	Rev	TGCACAGGAGCCAAGAGTGAA

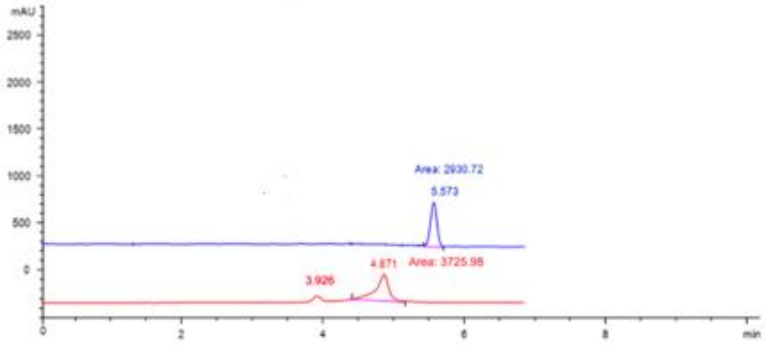

Figure S10. RP-HPLC copy of compounds **6** (blu) and **7** (red).

MATERIALS AND METHODS

GSK-3β inhibition. Kinase-Glo assays (Promega Biotech Iberica, SL) were performed in assay buffer using black 96-well plates. In a typical assay, 10 µl (10 µM) of test compound (dissolved in DMSO at 1 mM concentration and diluted in advance in assay buffer to the desired concentration) and 10 µL (20 ng) of enzyme were added to each well followed by 20 µl of assay buffer containing 50 µM substrate and 2 µM ATP. The final DMSO concentration in the reaction mixture did not exceed 1 %. After 30-min incubation at 30 °C the enzymatic reaction was stopped with 40 µL of Kinase-Glo reagent. Glow-type luminescence was recorded after 10 min using a FLUOstar Optima (BMG Labtechnologies GmbH, Offenburg Germany) multimode reader. The activity is proportional to the difference in the total and consumed ATP. The inhibitory activities were calculated on the basis of maximal activities measured in the absence of inhibitor. The IC_{50} was defined as the concentration of each compound that reduces a 50 % the enzymatic activity with respect to that without inhibitors. Five different concentrations by duplicate were used to determine the dose/response curve.

Cell viability. SH-SY5Y cells were seeded in a 96-well plate at 2×10^4 cells/well, incubated for 24 h and afterwards treated with different concentrations of curcumin-DEF hybrids 4-7, TDZD and DMF (1.25–40 µM) for 24 h at 37°C in 5 % CO_2. Cell viability, in terms of mitochondrial metabolic function, was evaluated by 3-(4,5-dimethylthiazol-2-yl)-2,5-diphenyltetrazolium bromide (MTT) test, as previously described (see Supporting Information for experimental details).[2]

Detection of phosphorylated GSK-3β kinase by Western blotting. SH-SY5Y cells were seeded in 60 mm dishes at 2×10^6 cells/dish, incubated for 24 h and subsequently treated with curcumin-DEF hybrids **5** and **6** (5 µM) for 1, 3 and 6 h at 37°C in 5 % CO_2. At the end of incubation, cells were lysed by the addition of ice-cold lysis buffer containing leupeptin 2 µg/mL and PMSF 100 µg/mL. An aliquot was used for protein analysis with the Bradford assay for protein quantification. Cell lysates (50 µg per sample) were separated by SDS-polyacrylamide gels and transferred onto nitrocellulose membranes, which were probed with primary phospho-GSK3α/β (Ser21/9) (1:1000; Cell Signaling Technology, Danvers, MA, USA) and secondary antibodies. ECL reagents (Pierce, Rockford, IL, USA) were utilized to detect targeted bands. The same

membranes were stripped and reprobed with GSK-3β (1:1000; Cell Signaling Technology) and β-Actin (1:1000; Sigma Aldrich) antibodies. Data were analysed by densitometry, using Quantity One software (Bio-Rad, Hercules, CA, USA). Data are expressed as a ratio between phospho-GSK3α/β and GSK3β levels normalized against β-Actin. Data were analysed by densitometry, using Quantity One software (Bio-Rad). Data are expressed as fold increase versus respective contralateral intact site.

BBB permeation. Prediction of the brain penetration was evaluated using a parallel artificial membrane permeability assay (PAMPA).[1] Ten commercial drugs, phosphate buffer saline solution at pH 7.4 (PBS), Ethanol and dodecane were purchased from Sigma, Acros organics, Merck, Aldrich and Fluka. The porcine polar brain lipid (PBL) (catalog no. 141101) was from Avanti Polar Lipids. The donor plate was a 96-well filtrate plate (Multiscreen® IP Sterile Plate PDVF membrane, pore size is 0.45 μM, catalog no. MAIPS4510) and the acceptor plate was an indented 96-well plate (Multiscreen®, catalog no. MAMCS9610) both from Millipore. Filter PDVF membrane units (diameter 30 mm, pore size 0.45 μm) from Symta were used to filtered the samples. A 96-well plate UV reader (Thermoscientific, Multiskan spectrum) was used for the UV measurements. Test compounds [(3-5 mg of Caffeine, Enoxacine, Hydrocortisone, Desipramine, Ofloxacine, Piroxicam, Testosterone), (12 mg of Promazine) and 25 mg of Verapamile and Atenolol] were dissolved in EtOH (1000 μL). 100 microlitres of this compound stock solution was taken and 1400 μL of EtOH and 3500 μL of PBS pH =7.4 buffer were added to reach 30% of EtOH concentration in the experiment. These solutions were filtered. The acceptor 96-well microplate was filled with 180 μL of PBS/EtOH (70/30). The donor 96-well plate was coated with 4 μL of porcine brain lipid in dodecane (20 mg mL^{-1}) and after 5 minutes, 180 μL of each compound solution was added. 1-2 mg of every compound to be determined their ability to pass the brain barrier were dissolved in 1500 μL of EtOH and 3500 μL of PBS pH=7.4 buffer, filtered and then added to the donor 96-well plate. Then the donor plate was carefully put on the acceptor plate to form a "sandwich", which was left undisturbed for 2h and 30 min at 25 °C. During this time the compounds diffused from the donor plate through the brain lipid membrane into the acceptor plate. After incubation, the donor plate was removed. UV plate reader determined the concentration of compounds and commercial drugs in the acceptor and the donor wells. Every sample was analyzed at three to five wavelengths, in 3 wells and in two independent runs. Results are given as the mean [standard deviation (SD)] and the average of the two runs is reported. 10 quality control compounds (previously mentioned) of known BBB permeability were included in each experiment to validate the analysis set.

References

1. Di, L.; Kerns, E. H.; Fan, K.; McConnell, O. J.; Carter, G. T. (2003) High throughput artificial membrane permeability assay for blood-brain barrier. *Eur. J. Med. Chem. 38,* 223-232.
2. Rampa, A.; Tarozzi, A.; Mancini, F.; Pruccoli, L.; Di Martino, R. M.; Gobbi, S.; Bisi, A.; De Simone, A.; Palomba, F.; Zaccheroni, N.; Belluti, F. (2016) Naturally Inspired Molecules as Multifunctional Agents for Alzheimer's Disease Treatment. *Molecules. 21,* 643.
